# Neoantigen peptide-pulsed dendritic cell vaccine therapy after surgical treatment of pancreatic cancer: a retrospective study

**DOI:** 10.3389/fimmu.2025.1571182

**Published:** 2025-04-03

**Authors:** Koki Oyama, Kohei Nakata, Toshiya Abe, Kento Hirotaka, Nao Fujimori, Kazuma Kiyotani, Chika Iwamoto, Naoki Ikenaga, Shinji Morisaki, Masayo Umebayashi, Hiroto Tanaka, Norihiro Koya, Shinichiro Nakagawa, Kenta Tsujimura, Sachiko Yoshimura, Hideya Onishi, Yusuke Nakamura, Masafumi Nakamura, Takashi Morisaki

**Affiliations:** ^1^ Department of Surgery and Oncology, Graduate School of Medical Sciences, Kyushu University, Fukuoka, Japan; ^2^ Department of Medicine and Bioregulatory Science, Graduate School of Medical Sciences, Kyushu University, Fukuoka, Japan; ^3^ Laboratory of Immunogenomics, Center for Intractable Diseases and ImmunoGenomics, National Institute of Biomedical Innovation, Health and Nutrition, Osaka, Japan; ^4^ Department of Cancer Immunotherapy, Fukuoka General Cancer Clinic, Fukuoka, Japan; ^5^ Department of Medicine and Clinical Science, Graduate School of Medical Sciences, Kyushu University, Fukuoka, Japan; ^6^ Corporate Headquarters, Cancer Precision Medicine Inc., Kawasaki, Japan

**Keywords:** neoantigen, DC vaccine, pancreatic cancer, PDAC, immunotherapy

## Abstract

**Introduction:**

Pancreatic cancer shows very poor prognosis and high resistance to conventional standard chemotherapy and immunotherapy; therefore, the development of new breakthrough therapies is highly desirable.

**Method:**

We retrospectively evaluated the safety and efficacy of neoantigen peptide-pulsed dendritic cell (Neo-P DC) vaccine therapy after surgical treatment of pancreatic cancer.

**Result:**

The result showed induction of neoantigen-specific T cells in 13 (81.3%) of the 16 patients who received Neo-P DC vaccines. In survival analysis of the nine patients who received Neo-P DC vaccines after recurrence, longer overall survival was observed in patients with neoantigen-specific T cell induction than those without T cell induction. Notably, only one of the seven patients who received Neo-P DC vaccines as adjuvant setting developed recurrence, and no patient died during median follow-up 61 months after surgery (range, 25-70 months). Furthermore, TCR repertoire analyses were performed in a case treated with Neo-P DC vaccine combined with long and short peptides, and one significantly dominant clone induced by the long peptide was detected among CD4^+^ T cell populations.

**Discussion:**

The present study suggests the feasibility and efficacy of Neo-P DC vaccine therapy after surgical treatment of pancreatic cancer in both postoperative recurrence cases and adjuvant setting. A case analysis suggests the importance of combination with long peptides targeting CD4^+^ T cell.

## Introduction

Pancreatic cancer shows very poor prognosis with a 5-year survival rate of 13% due to diagnosis at advanced stages and high resistance to standard chemotherapy (1). With the further increase of morbidity and no significant improvement of survival rates, pancreatic cancer is estimated to become the second leading cause of cancer-related death by 2030 (2, 3). Although immune checkpoint inhibitors have provided a new treatment option for cancer (4, 5), their therapeutic effects in pancreatic cancer are very limited because of the lower mutation burden in cancer cells as well as immune-suppressive tumor microenvironment (TME) (6, 7). However, researches have shown that patients who have pancreatic cancer with high microsatellite instability responded exceptionally well to immune checkpoint inhibitors, and that long survivors might have neoantigen-stimulated T cells (8, 9). These reports suggest that induction of neoantigen-specific T cells is likely to contribute to establishment of more effective antitumor immunotherapy, even for patients with pancreatic cancer.

The cancer genome analysis combined with next-generation sequencing technologies has enabled personalized medicine based on patient-specific somatic-mutation that can be applied to predict the optimal neoantigens that have high binding-affinity to human leukocyte antigen (HLA) molecules (10). We previously described a neoantigen prediction pipeline for the design of personalized therapeutic neoantigen vaccines (11). Others reported that personalized therapeutic cancer vaccines composed of neoantigens are feasible, safe, and immunogenic in patients with melanoma and glioblastoma (12–16). The efficacy of personalized RNA neoantigen vaccines as a postoperative adjuvant setting was recently reported in pancreatic cancer, which is thought to be a representative “immunologically cold” tumor (17). However, the best way to deliver vaccines (e.g., peptides, RNA, DNA, or viral vectors with/without dendritic cells [DCs]), a combination with other therapies, and significance of long peptides inducing CD4^+^ T cells are still under discussion.

Based on the safety of DC administration, which has been clarfied in many clinical trials (18, 19), and the efficacy of neoantigen peptides, we initiated neoantigen peptide-pulsed DC (Neo-P DC) vaccine therapy for various solid tumors in accordance with the Japanese Regenerative Medicine Safety Assurance Act (20–23). We herein describe the results of a retrospective study on the efficacy and clinical application of Neo-P DC vaccine therapy after surgical treatment of pancreatic cancer.

## Materials and methods

### Patient characteristics and Neo-P DC vaccine therapy

This study complies with the Declaration of Helsinki. Neoantigen analysis and Neo-P DC vaccine therapies at our institute have been approved by the Ethics Committee of Fukuoka Cancer Clinic (FGCC-EC009) as with our previous study (22). All patients provided written informed consent for the procedure based on the requirements for class III regenerative medicine under the Japanese Act on the Safety of Regenerative Medicine. This study is a retrospective analysis limited to pancreatic cancer cases among those patients who received Neo-P DC vaccine therapies under the ethical approvement at the institute. The group was comprised of 16 patients who underwent Neo-P DC vaccine therapy between November 2018 and November 2022 at Department of Cancer Immunotherapy, Fukuoka General Cancer Clinic (Fukuoka, Japan) after pancreatectomy. Pancreatectomies were performed between January 2016 and November 2021.

**Figure 1:**
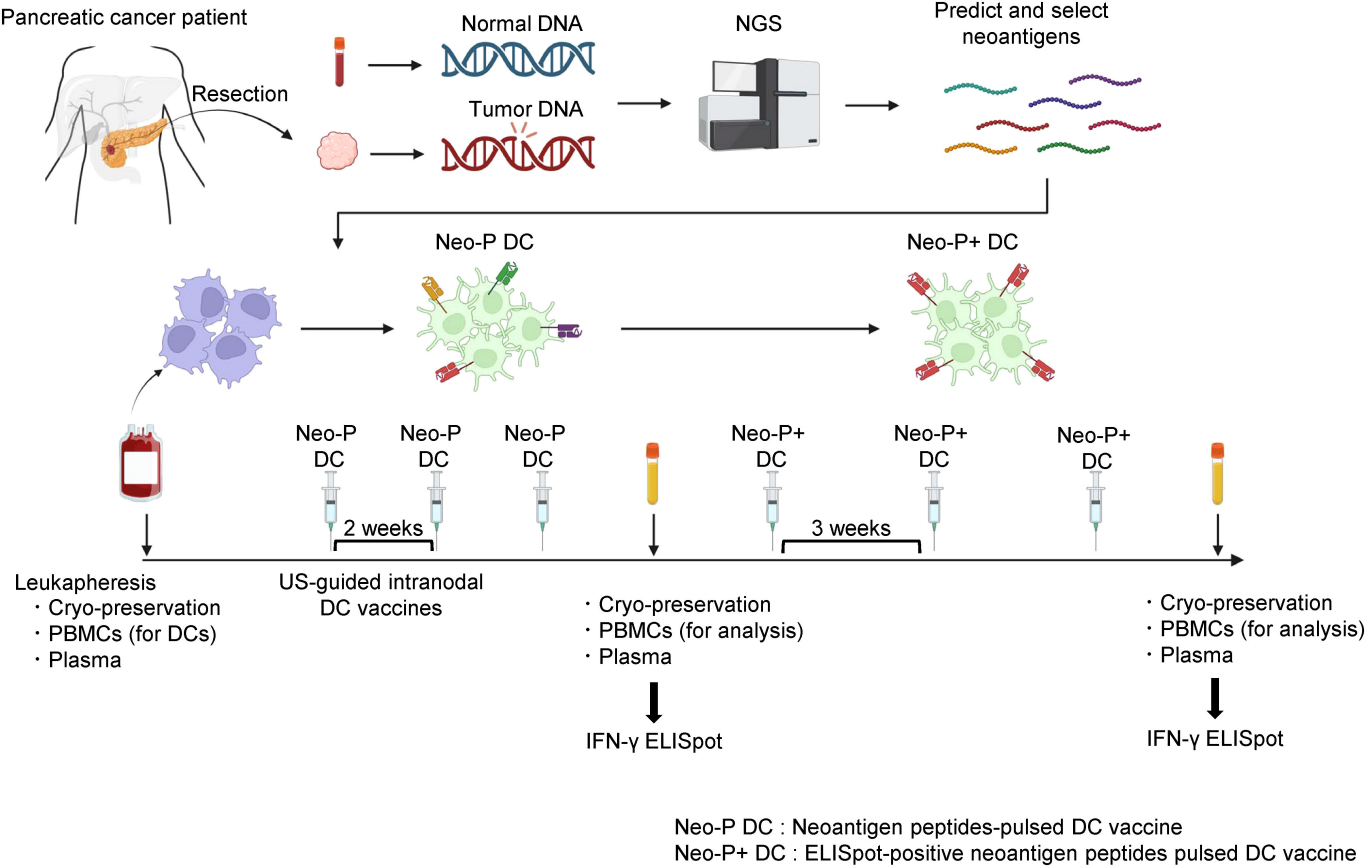
Protocol for intranodal Neo-P DC vaccine therapy. Predicted neoantigens based on single-nucleotide variants identified by NGS were synthesized, and DCs differentiated from patient-derived PBMCs were pulsed by synthesized neoantigen peptides to generate Neo-P DC vaccines. The Neo-P DC vaccines were directly administered into the lymph nodes under US guidance. After the administration of three vaccines at 2-week intervals, IFN-γ ELISpot analysis was performed, and Neo-P+ DCs were used for the following three vaccines. After administering the six DC vaccines, ELISpot responses were measured again. PBMCs and plasma were cryopreserved before, during, and after treatment. NGS, next-generation sequencing; DCs, dendritic cells; PBMCs, peripheral blood mononuclear cells; US, ultrasound; IFN, interferon; ELISpot, enzyme-linked immunospot; Neo-P DC, neoantigen peptide-pulsed dendritic cell; NeoP+ DC, IFN-γ ELISpot-positive neoantigen peptide-pulsed dendritic cell.

**Figure 1** shows the protocol for Neo-P DC vaccine therapy. The first three vaccinations were performed at 7- to 14-day intervals by ultrasound-guided intranodal injection. After the third vaccination, peripheral blood mononuclear cells (PBMCs) were isolated to examine the immune response to each neoantigen peptide using interferon-γ (IFN-γ)-based enzyme-linked immunospot (ELISpot) responses. The next three vaccinations were administered three times at 3-week intervals, and tumor responses were assessed with computed tomography (CT) after completion. PBMCs and plasma were collected before treatment, during vaccine therapy, and after completion of the six vaccinations.

### Neoantigen prediction

We used the neoantigen prediction as previously described (11, 22). Briefly, the patients’ HLA class I and class II genotypes were estimated from whole-exome sequencing data of peripheral blood using the computational tools OptiType (24) and PHLAT (25). NetMHC v3.4 and NetMHCpanv2.8 were used for the estimation of the binding affinities of short (8- to 11-mer) peptides for HLA-A and HLA-B as previously described (11, 26). NetMHCII-2.2 and netMHCIIpan-3.1 were used for the estimation of the binding affinities of long (15- to 18-mer) peptides for HLA-DRB1 (26, 27). Peptides with binding affinity (IC50) less than 50 nM were selected as candidate neoantigens. The Cancer Genome Atlas mRNA expression data for pancreatic ductal adenocarcinoma was combined to select potential neoantigen candidates. **Supplementary Tables 1**–**3** show the list of neoantigens selected in each case.

### Establishment and administration of DC vaccine

DC vaccines were established as previously described (20–23). Briefly, cryopreserved PBMCs obtained by pretreatment leukapheresis were thawed and seeded (2 × 10^6^ mononuclear cells/well) in six-well plates. After removing the supernatant including floating cells, adherent cells were cultured in DC complete medium containing 100 ng/mL granulocyte-macrophage colony-stimulating factor (Primmune Inc., Kobe, Japan) and 50 ng/mL Interleukin (IL)-4 (Primmune Inc.). On day 6, two maturation factors, 500 IU/mL tumor necrosis factor-α (TNF-α) (PeproTech Inc., Cranbury, NJ, USA) and 500 IU/mL IFN-α (Sumitomo Pharma, Osaka, Japan), were added to induce mature DCs. Fluorescence-activated cell sorting analysis revealed that the cells were positive for DC maturation markers (HLA-DR, HLA class I, CD86, and CD40) and negative for a monocyte marker (CD14), confirming differentiation to mature DCs. Before use, the neoantigen peptides were proved negative for endotoxin, β-glucan, and *Mycoplasma* using Toxinometer ET-6000 (Wako Pure Chemical Industries, Ltd., Osaka, Japan) and *Mycoplasma* detection assay (MycoAlert; Lonza Rockland Inc., Rockland, ME, USA). The Short peptides for class I were introduced to matured DCs, and long peptides for class II were introduced pre-matured DCs. Peptide-pulsed DCs were suspended in 500 µL saline and administered to the corticomedullary border of normal inguinal lymph nodes using a 25-G needle under ultrasound guidance, as reported in detail in our previous papers (18, 19, 22).

### IFN-γ ELISpot response

Human IFN-γ ELISpot Plus Kit (Mabtech Inc., Cincinnati, OH, USA) was used as previously described (22). Briefly, DCs derived from frozen PBMCs cryopreserved before therapy were spread onto 96-well ELISpot plates precoated with anti-IFN-γ antibody at a density of 5 × 10^3^ cells/well. DC maturation was performed as described above section. Short peptides were added to matured DC, and long peptides were added to pre-matured DC. Lymphocytes isolated from the cryopreserved PBMCs obtained before and after vaccine therapy were co-cultured with neoantigen-pulsed DCs at 1.5 × 10^5^ cells/well for 48 h. After the detection antibody (7-B6-1-biotin, 1 µg/mL, 100 µL) was added and incubated for 2 h, the secondary antibody (1 µg/mL, 100 µL) was added and incubated for 1 h. After 100 mL/well of TMB substrate solution was added for 10 min, the spots were analyzed using an Automated ELISpot Reader 0.8 Classic (AID GmbH, Strasberg, Germany).

### Enrichment and expansion of neoantigen-reactive T lymphocytes

Monocyte-derived immature DCs from a patient with recurrence (Recurrence #8) were pulsed with a long peptide (NGKKVRKNWLSSWAVLQV; Peptide No. 6 in **Supplementary Table 3**), and DC maturation factors (TNF-α + IFN-α) were then added to induce neoantigen-pulsed mature DCs. These neoantigen-pulsed DCs were then co-cultured with lymphocytes collected after administration of Neo-P DC vaccines at a ratio of 1:20 for 2 weeks in complete medium supplemented with low-dose 20 U/mL human recombinant IL-2 (Primmune Inc.). The neoantigen-pulsed DCs were then added to the lymphocytes again, restimulated, and cultured for another 2 weeks to expand the neoantigen-specific T lymphocytes.

### T-cell receptor sequencing analysis

TCR sequencing was performed using previously described methods (28, 29). In brief, we used total RNA extracted from 2 × 10^6^ T cells for cDNA synthesis with a common 5′-RACE adapter using the SMART Library Construction Kit (Clontech Laboratories, Mountain View, CA, USA). TCRα and TCRβ cDNAs were amplified by PCR using a forward primer for the SMART adapter and a reverse primer corresponding to the constant region of the TCRα and TCRβ genes. Illumina index sequences with barcodes were added using the Nextera XT Index kit (Illumina, San Diego, CA, USA), and the prepared libraries were sequenced by 300-bp paired-end reads on the Illumina MiSeq System using MiSeq Reagent v3 600-cycles kit (Illumina). Sequence data were analyzed using Tcrip software. (28) The inverse Simpson’s diversity index and the clonality based on the Shannon index in the context of CDR3 sequences were used to evaluate TCR clonality.

### Immunohistochemistry

Paraffin-embedded pancreatic tissues were sliced into 4-µm-thick sections. The sections were deparaffinized in xylene and rehydrated using an ethanol gradient. Antigen retrieval was achieved in sodium citrate buffer (pH 6.0) or Tris-EDTA buffer (pH 9.0) using a pressure cooker. After blocking with 3% bovine serum albumin in PBS, the sections were incubated with the primary antibody anti-CD20 (1:200, L26 antibody, #422741; Nichirei Biosciences, Tokyo, Japan) or anti-HLA-DR (1:250, CR3/43, #MA1-25914, AB_794857; Invitrogen, Waltham, MA, USA) diluted in 1% bovine serum albumin overnight at 4°C. Next, the sections were labeled with EnVision+ System HRP-labeled polyclonal anti-rabbit antibody (#K4003; Dako, Glostrup, Denmark) or anti-mouse antibody (#K4001; Dako) for 1 h at room temperature and visualized with a 3,3′-diaminobenzidine kit (#D5537-5G; Sigma-Aldrich, St. Louis, MO, USA). Counterstaining was performed with hematoxylin. Images were acquired on a fluorescence microscope (BZ-X800; Keyence, Osaka, Japan).

### Statistical analysis

All statistical analyses and graph generation were performed using GraphPad Prism ver.9.3.1 (GraphPad Software, La Jolla, CA, USA). Survival analyses were conducted using the Kaplan–Meier method, and the curves were compared using the log-rank test.

## Results

### Treatment with Neo-P DC vaccine

We administered Neo-P DC vaccines to postoperative 16 patients with pancreatic cancer. **Tables 1**, **2** show the clinical characteristics of the patients in this study; nine patients with postoperative recurrence (**Table 1**) and seven patients who received as adjuvant setting after pancreatectomy (**Table 2**).

**Table 1:** Clinical characteristics of patients (Post-operative recurrence cases).

#	Age/ Gender	Surgery	NAC	pStage	Adjuvant	Rec site	Post-rec Therapy	Post-ope RFS	Survival	Post-ope OS	Post-rec OS
1	52/F	PD+PVR	(-)	IB	S-1	LYM, PER	GEM, RT, GnP	5M	Alive	68M	63M
2	71/F	SSPPD	GnP	IIB	S-1	HEP, PUL	GnP	6M	Dead	16M	10M
3	42/M	DP+Spl	(-)	III	S-1	PUL	GnP, mFFX	2M	Dead	30M	28M
4	75/F	PPPD	(-)	III	S-1	LYM, PER, PUL	GnP	7M	Alive	93M	86M
5	72/F	DP+Spl	(-)	IIB	S-1	HEP, PER	GnP, mFFX	11M	Dead	40M	29M
6	56/M	DP;Spl	(-)	IB	(-)	PUL	(-)	21M	Dead	53M	32M
7	66/M	SSPPD	(-)	IIB	S-1	LYM	GnP	3M	Alive	40M	37M
8	72/M	PPPD	GS	III	S-1	LYM	mFFX	26M	Alive	46M	20M
9	67/M	PPPD	(-)	IIB	(-)	LYM	(-)	6M	Dead	19M	13M

NAC, neoadjuvant chemotherapy; Rec site, recurrence site, Post-rec, post-recurrence; Post-ope, post-operative; RFS, recurrence-free survival; OS, overall survival; PD, pancreatoduodenectomy; PVR, portal vein resection; SSPPD, subtotal stomach-preserving PD; DP, distal pancreatectomy; Spl, splenectomy; PPPD, pylorus-preserving PD; LYM, lymph node metastasis; PER, peritoneal dissemination; HEP, hepatic metastasis; PUL, pulmonary metastasis; RT, radiation therapy; GnP, GEM+nab-PTX; mFFX, modified FOLFIRINOX.

**Table 2:** Clinical Characteristics of Patients (Adjuvant cases).

#	Age/ Gender	Surgery	NAC	pStage	Adjuvant	Recurrence	Post-ope RFS	Survival	Post-ope OS
1	67/M	SSpPD+PVR	GnP	IIB	S-1	(-)	62M	Alive	62M
2	69/M	DP+Spl	(-)	IIB	S-1	HEP	25M	Alive	65M
3	66/F	PpPD	GnP	IIB	S-1	(-)	57M	Alive	57M
4	52/F	DP+Spl	(-)	IA	S-1	(-)	70M	Alive	70M
5	64/M	DP+Spl	(-)	IIA	S-1	(-)	61M	Alive	61M
6	61/M	PD	(-)	IA	GS	(-)	49M	Alive	49M
7	72/M	SSpPD	GS	IIB	S-1	(-)	25M	Alive	25M

Among the nine patients with postoperative recurrence, the pathological stage at the time of surgery was I, II, and III in two, four, and three patients, respectively. Among the seven patients who received Neo-P DC vaccine as adjuvant setting, two were in pathological stage I and five were in stage II. All vaccine therapies were administered with the same protocol (**Figure 1**). The Neo-P DC vaccine was generally administered concurrently with systemic chemotherapy. Profiles of neoantigens in each patient are listed in **Tables 3**, **4**; **Supplementary Tables 1**–**3** show the list of neoantigens selected in each case.

**Table 3:** Profiles of Neoantigens and Immunological Responses (Post-operative recurrence cases).

#	Number of SNVs	Number of Neoantigens	Number of Candidate Neoantigens (IC50<50nM)	Number of Selected Neoantigens (short)	Number of Selected Neoantigens (long)	Number of ELISpot-positive Peptides (short)	Number of ELISpot-positive Peptides (long)
1	46	44	3	13	(-)	4	(-)
2	33	99	5	8	(-)	0	(-)
3	42	73	1	12	(-)	0	(-)
4	141	47	2	5	(-)	2	(-)
5	49	90	5	5	(-)	2	(-)
6	99	351	24	10	(-)	4	(-)
7	23	82	2	4	3	2	1
8	46	115	2	5	4	0	1
9	29	150	2	5	4	3	0

SNV, single nucleotide variant; IC50, half-maximal inhibitory concentration.

**Table 4:** Profiles of Neoantigens and Immunological Responses (Adjuvant cases).

#	Number of SNV	Number of Neoantigens	Number of Candidate Neoantigens (IC50<50nM)	Number of Selected Neoantigens (short)	Number of Selected Neoantigens (long)	Number of ELISpot-positive Peptides (short)	Number of ELISpot-positive Peptides (long)
1	10	36	3	3	(-)	1	(-)
2	99	87	3	7	(-)	2	(-)
3	20	25	1	10	(-)	3	(-)
4	10	36	2	8	(-)	2	(-)
5	7	14	1	4	(-)	1	(-)
6	14	51	1	6	3	3	1
7	26	64	1	4	2	3	1

### Immune responses after Neo-P DC vaccine administration

**Figure 2:**
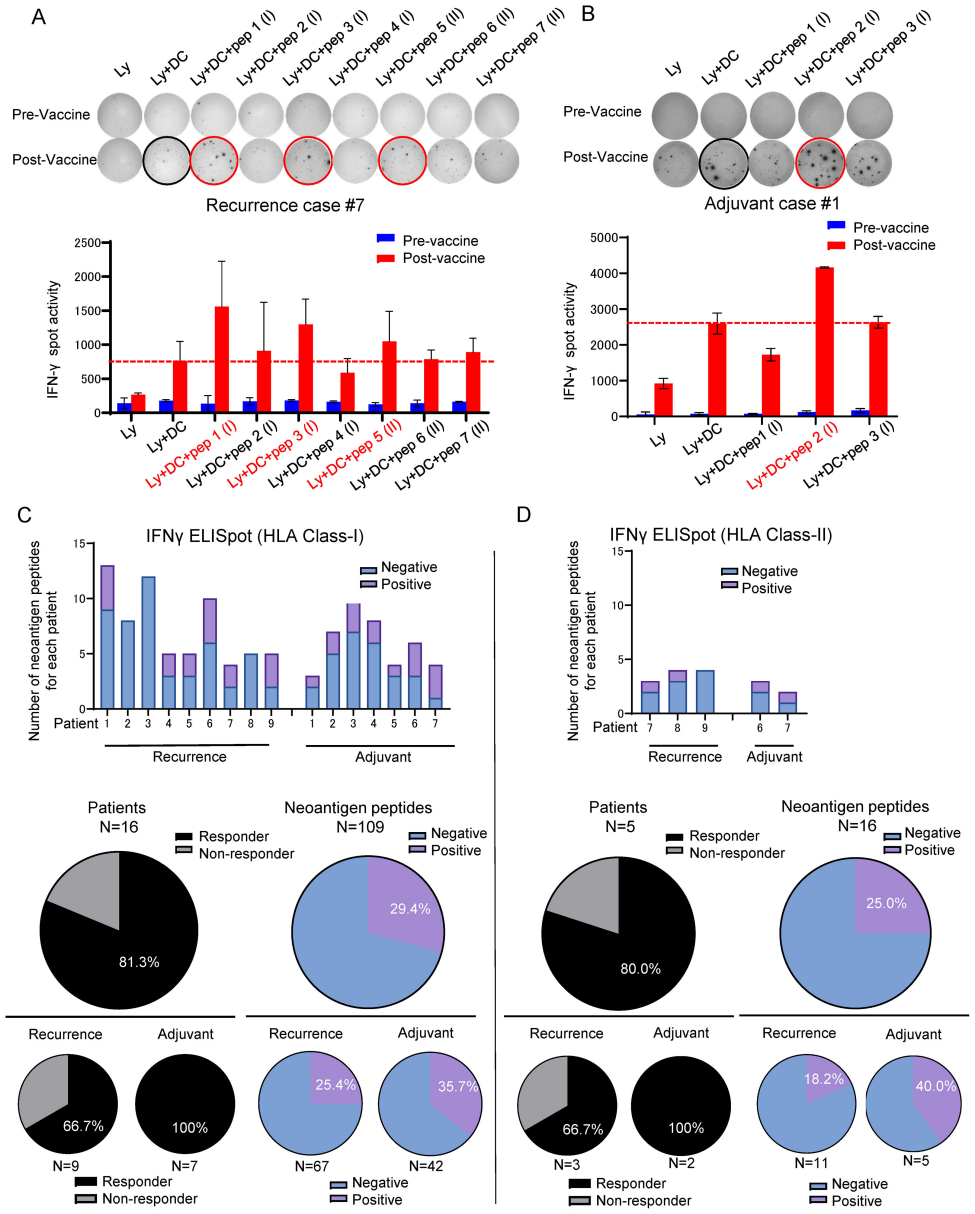
Neoantigen-pulsed DC vaccine reliably induced neoantigen-specific T cells. Immune responses of peripheral blood lymphocytes to neoantigen peptides before and after Neo-P DC vaccine therapy in **(A)** Recurrence Case #7 and **(B)** Recurrence Prevention Case #1. Each panel shows the IFN-γ ELISpot response to neoantigen peptide measured in peripheral blood lymphocytes from each patient [Ly, lymphocytes alone; Ly + DC, lymphocytes + dendritic cells; Ly + DC + pep (I), lymphocytes + dendritic cells + neoantigen peptide I (class I peptide); Ly + DC + pep (II), lymphocytes + dendritic cells + neoantigen peptide II (class II peptide). **(C)** (Upper) Results of ELISpot responses to class I peptides in all 16 cases. (Middle) Percentages of responders and ELISpot-positive peptides among all 109 class I peptides. (Lower) Percentages in each case of recurrence and adjuvant therapy. **(D)** (Upper) Results of ELISpot responses to class II peptides in all five cases. (Middle) Percentages of responders and ELISpot-positive peptides among all 16 class II peptides. (Lower) Percentages in each case of recurrence and adjuvant therapy. ELISpot, enzyme-linked immunospot.

After the third DC vaccine administration, lymphocytes isolated from the patient’s peripheral blood were co-cultured with DCs and the neoantigen peptides used for the treatment, and the induction of neoantigen-specific T cells by the Neo-P DC vaccine was assessed using IFN-γ ELISpot assay (**Supplementary Figures S1**, **S2**). **Figures 2A, B** show representative ELISpot images; lymphocytes co-cultured with DCs without peptides were used as the control. Neoantigen-reactive T cells for short peptides were induced in 13 (81.3%) of the 16 patients, and notably, in all 7 patients who were treated as the adjuvant setting, neoantigen-specific T cells were induced against at least one peptide (**Figure 2C**). Among 109 short peptides, we observed specific T cell induction in 32 peptides (29.4%) by ELISPOT assay (**Figure 2C**). Among 67 short peptides used in patients with postoperative recurrence, 17 peptides (25.4%) successfully induced neoantigen-specific T cells; while among 42 short peptides in the patients treated as adjuvant setting, 15 peptides (35.7%) induced neoantigen-specific T cells. Furthermore, we applied long neoantigen peptides, which we expected to induce neoantigen-specific CD4^+^ T cells, in five recently treated patients. Induction of neoantigen-reactive T cells to these long peptides were observed in four of the five patients (**Figure 2D**). Among the 16 long peptides, 4 peptides (25.0 %) successfully induced neoantigen-specific T cells (**Figure 2D**). Among 11 long peptides used in postoperative recurrence cases, 2 peptides (18.2%) successfully induced neoantigen-specific T cells; while among 5 long peptides in the patients treated as adjuvant setting, 2 peptides (40.0%) induced neoantigen-specific T cells.

### Retrospective prognostic analysis of 16 patients with pancreatic cancer treated with Neo-P DC vaccine

**Figure 3:**
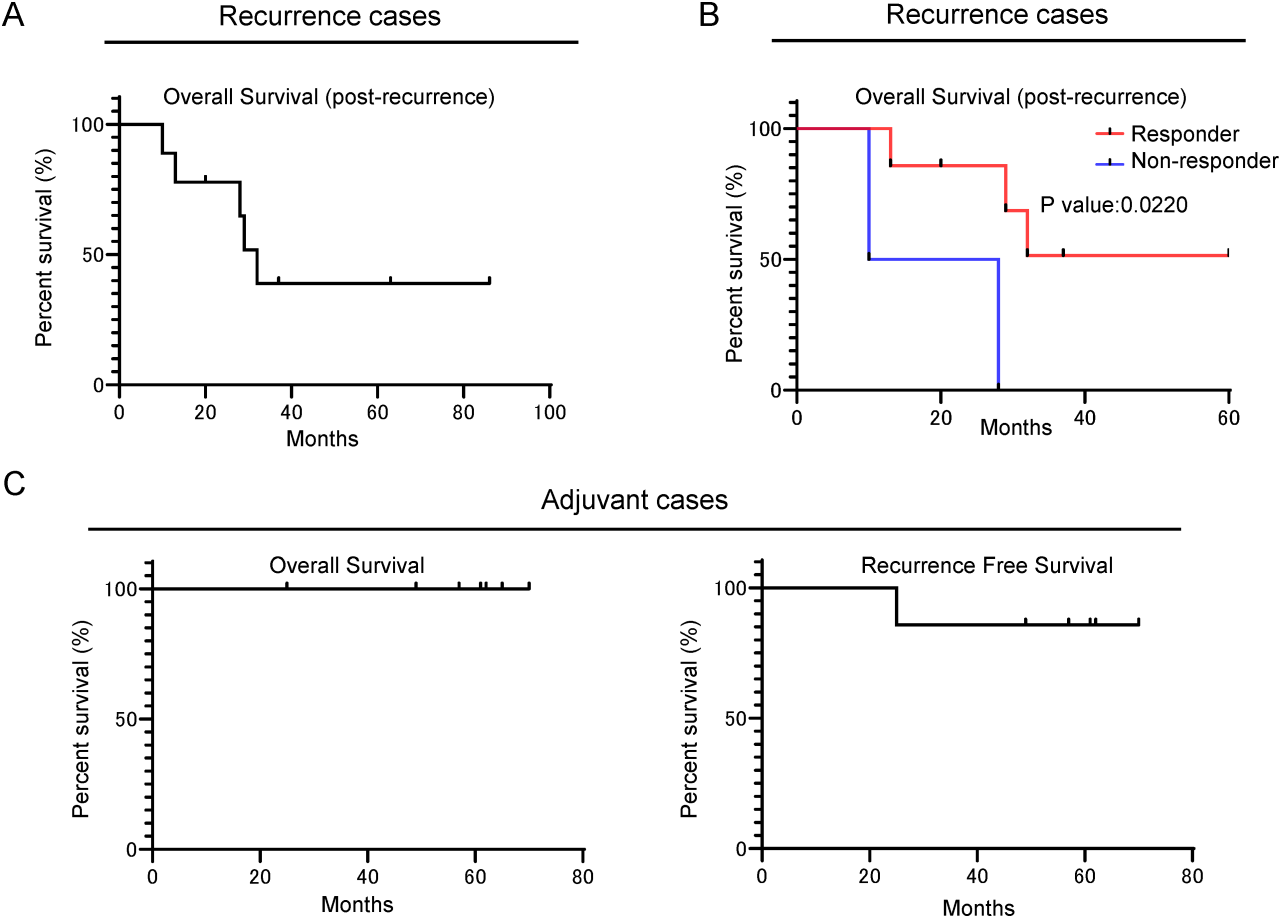
Retrospective survival analysis showing that Neo-P DC vaccine therapy can be expected to improve the prognosis of patients with pancreatic cancer. **(A)** Kaplan–Meier OS analysis (post-recurrence OS) of patients with recurrence treated with Neo-P DC vaccine therapy. **(B)** Kaplan–Meier OS analysis (post-recurrence OS) of responders and non-responders to Neo-P DC vaccines among patients with recurrence. **(C)** Kaplan–Meier survival analysis of OS and recurrence-free survival of postoperative patients treated with Neo-P DC vaccines for prevention of recurrence. Neo-P DC, neoantigen peptide-pulsed dendritic cell; OS, overall survival.

We examined patient survival after Neo-P DC vaccine therapy. Among the nine patients, who received vaccination after postoperative recurrence, three patients achieved over 36 months survival after recurrence (**Figure 3A**). Furthermore, the seven patients who showed the induction of neoantigen-specific T cells by the Neo-P DC vaccine revealed better prognosis than non-responders (**Figure 3B**).

All seven patients who received Neo-P DC vaccine therapy as adjuvant setting were alive during the median follow-up period of 61 months (range, 25–70 months), although one case had recurrence (**Figure 3C**). This recurrence case involved a single lesion of hepatic metastasis that disappeared by radiofrequency ablation. The patient has shown no new sign of recurrence for 50 months after the radiofrequency ablation therapy.

### Efficacy of T-cell induction by long peptide binding to MHC II (Case Report)

**Figure 4:**
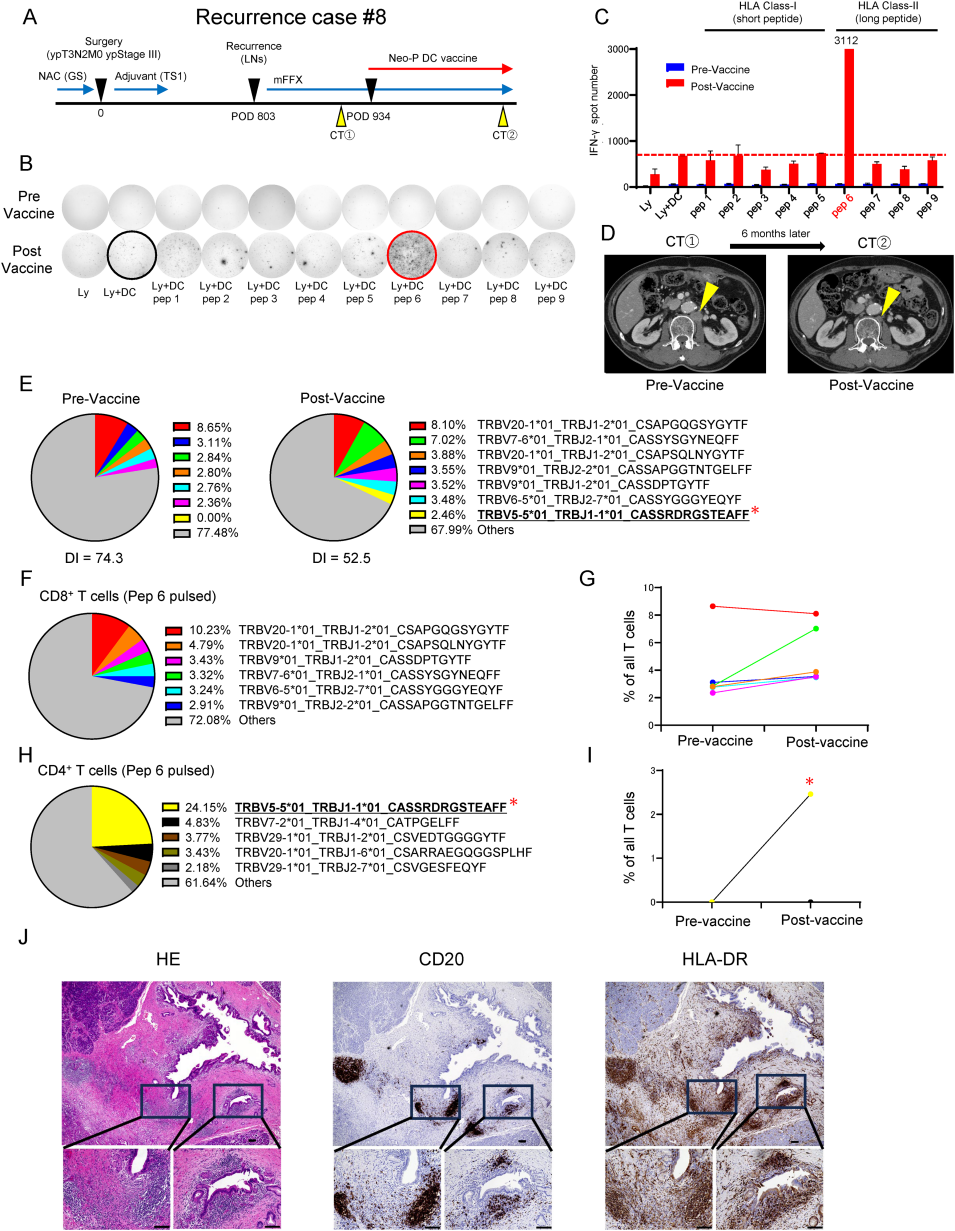
Recurrence Case #8 treated with long-peptide-combined Neo-P DC vaccine shows the importance of neoantigen-specific CD4^+^ T cells. **(A)** Recurrence Case #8. **(B)** Immune responses of peripheral blood lymphocytes to both short neoantigen peptides (class I peptides) and long neoantigen peptides (class II peptides) in Recurrence Case #8. The panel shows the IFN-γ ELISpot response to neoantigen peptides measured in peripheral blood lymphocytes [Ly, lymphocytes alone; Ly + DC, lymphocytes + dendritic cells; Ly + DC + pep **(I)**, lymphocytes + dendritic cells + peptide]. **(C)** Graph of IFN-γ spot number in **(B)**. Note the marked responses of T cells to class II affinity long neoantigen (pep 6). **(D)** Computed tomography image showing the change in metastatic lesion before and after Neo-P DC vaccine therapy. **(E)** Analysis of TCRβ repertoire for pre- and post-vaccine T cells in peripheral blood. The top six most frequent clones are listed in pre-Vaccine, and the top seven most frequent clones are listed in post-Vaccine. DI, inverse Simpson’s diversity index. **(F)** Analysis of TCRβ repertoire for CD8^+^ T cells sorted from post-vaccine peripheral blood mononuclear cell–derived lymphocytes expanded by being pulsed with pep 6. The top six most frequent clones are listed. **(G)** Frequency of the top six enriched CD8^+^ T cell clones **(F)** in pre- and post-vaccine peripheral blood. **(H)** Analysis of TCRβ repertoire for CD4^+^ T cells sorted from post-vaccine peripheral blood mononuclear cell–derived lymphocytes expanded by being pulsed with pep 6. The top five most frequent clones are listed. **(I)** Frequency of the top five enriched CD4^+^ T cell clones **(H)** in pre- and post-vaccine peripheral blood. **(J)** Immunohistochemical analysis of primary tumor (Recurrence Case #8) for HE, CD20 (B-cell marker), and HLA-DR. Scale bars, 100 µm. IFN-γ ELISpot, interferon-γ–based enzyme-linked immunospot; Neo-P DC, neoantigen peptide-pulsed dendritic cell; TCR, T-cell receptor. * in **(E, H, I)** refers to the most expanded clone by pulsed with pep6.

While most of the neoantigen vaccine therapies targeted the priming of neoantigen-specific CD8^+^ T cells using HLA class I-binding short peptides, the importance of neoantigen-specific CD4^+^ T cells has recently received attention (30–32). We have therefore attempted to use long peptides that are likely to bind to HLA class II molecules in combination with short peptides in several recent cases. In one case, a patient with recurrence (Recurrence #8), in whom a combination of long peptides and short peptides was used, revealed very strong induction of neoantigen-reactive T cells. The patient underwent radical pylorus-preserving pancreaticoduodenectomy for pancreatic head cancer following neoadjuvant chemotherapy with gemcitabine + S-1 and was diagnosed with ypT3N2M0 ypStage III with metastasis in 6 of 71 lymph nodes examined. After postoperative adjuvant chemotherapy with S-1, a CT scan showed an enlarged para-aortic lymph node metastasis on postoperative 26 months. Systemic chemotherapy, modified FOLFIRINOX (mFFX), was applied, but a 3-month post-therapy CT scan showed no change in tumor size. After that, Neo-P DC vaccine therapy was started in combination with modified mFFX on postoperative 31 months (**Figure 4A**). We selected neoantigen peptides, 5 short and 4 long peptides, from a list of 46 somatic non-synonymous mutations. The IFN-γ ELISpot assay revealed that while no short peptide significantly induced neoantigen-reactive T cells, one long peptide (pep 6) prominently induced neoantigen-reactive T cells (**Figures 4B, C**). After completion of six Neo-P DC vaccinations, CT showed remarkable shrinkage of the metastasis (**Figure 4D**). In addition, TCR repertoire analysis of pre- and post-vaccine peripheral blood showed clonal expansion of T cells, suggesting that specific T-cell clones were induced by Neo-P DC vaccines (**Figure 4E**). To determine which T-cell clone(s) was induced by pep 6 in this case, we analyzed post-vaccinated lymphocytes by fluorescence-activated cell sorting analysis and found that CD4^+^ T cells were enriched. Then, TCR repertoire analyses of sorted CD4^+^ T cells and CD8^+^ T cells were performed, and one significantly dominant clone was detected among CD4^+^ T cell populations (**Figures 4F–I**). The dominant clone induced by pep 6 was below the detection limit in the pre-vaccination peripheral blood, whereas it was detected at a frequency of 2.5% in the post-vaccination peripheral blood (**Figure 4I**). Analysis of CD8^+^ T cells pulsed with pep 6 revealed that the most frequent clones had been enriched before administration of the vaccine, and we concluded that they were vaccine-independent clones (**Figure 4G**). Overall, these results might suggest that the long neoantigen peptide induced neoantigen-specific CD4^+^ T cells and contributed to tumor shrinkage.

Building on the findings that showed the contribution of neoantigen-specific CD4^+^ T cells, we performed immunohistochemical analysis of HLA class II expression in primary tumors. Unexpectedly, the cancer cells did not significantly express HLA-DR; however, the aggregation of HLA-DR-expressing cells was observed, and theses populations were revealed to be B cells by expression of CD20 (**Figure 4J**).

Unfortunately, metastasis to Virchow’s lymph node subsequently appeared; at the time of this writing, however, the patient was receiving the second Neo-P DC vaccine therapy based on the neoantigen analysis obtained from the new metastasis.

## Discussion

In the present study, we retrospectively examined the scientific rationale and efficacy of Neo-P DC vaccine therapy after surgical treatment of pancreatic cancer. As the safety of DC vaccine therapy has already been widely reported in clinical practice (18, 19, 33), no patients in this study developed any adverse reactions, and all patients were able to complete the six courses of Neo-P DC vaccine therapy.

Among the 109 short peptides and 16 long peptides, neoantigen-reactive T cells were successfully induced for 32 short peptides (29.4%) and 4 long peptides (25.0%). 13 of the 16 patients acquired at least one neoantigen-reactive T cell (including both for short peptides and for long peptides) through Neo-P DC vaccines. The seven patients who received Neo-P DC vaccines as adjuvant setting tended to respond better to vaccinations (35.7% of short peptides and 40.0% of long peptides) than the 9 patients who received vaccinations after postoperative recurrence (25.4% of short peptides and 18.2% of long peptides), implying that vaccinations at an earlier stage would induce higher immune responses than those at advanced stages.

In our retrospective analysis of the nine patients who received Neo-P DC vaccines after postoperative recurrence, seven patients who showed the induction of at least one neoantigen-specific T cell by vaccinations revealed better prognosis than non-responders. This result indicates that the induction of neoantigen-specific T cells by the Neo-P DC vaccine is associated with the prognosis. Remarkable, among these seven responders, three patients have achieved survival of more than 36 months after postoperative recurrences. Considering previous reports that a median OS of the patients with postoperative recurrent pancreatic cancer who received standard systemic chemotherapies was 14 months (34), and the 5-year overall survival rate after surgical treatment of pancreatic cancer is only 30% to 40% (35–37), the Neo-P DC vaccine may improve the postoperative prognosis of patients with pancreatic cancer. Given the lack of efficacy of current standard chemotherapy alone for recurrent pancreatic cancer, our results suggest the efficacy of early postoperative induction with the Neo-P DC vaccine for patients with postoperative recurrence.

Among the seven patients who received Neo-P DC vaccines as adjuvant setting, induction of neoantigen-specific T cells was confirmed in all seven patients, and except for one patient with a single small liver metastasis, all six remaining patients had no recurrence. A previous prospective study also revealed the safety and efficacy of individualized neoantigen vaccine therapy using mRNA as adjuvant setting for pancreatic cancer, and they reported that all responders (8 of 16 patients, 50%) progressed without recurrence, whereas more than half of the non-responders developed recurrence within 1 year after surgery (17). These results suggest that while a drastic improvement in the prognosis can be expected if neoantigen-specific T cells are successfully induced by vaccination, the challenge of establishing an effective platform to reliably induce these T cells is a major issue that needs to be addressed. Although it is difficult to compare the superiority between neoantigen mRNA vaccine therapy and Neo-P DC vaccine therapy, the intranodal injection therapy with neoantigen peptide-pulsed DCs might be equivalent or superior to the use of mRNA in terms of inducing neoantigen-specific T cells.

Recent studies have revealed that DC paucity and dysfunction hamper effective immune surveillance against cancer cells and contribute to formation of the immunosuppressive TME observed in pancreatic cancer (38, 39). It has also been reported that cancer-associated fibroblasts, which are abundant in pancreatic cancer, inhibit differentiation of DCs and their function as antigen-presenting cells by secreting Wnt2 (40). Thus, a DC-based vaccination strategy using matured DCs pulsed with neoantigen peptides *ex vivo* may be more appropriate for pancreatic cancer. Various vaccine formulations and delivery strategies are currently being tested in clinical studies for various tumor types (41). However, we believe that there are significant advantages to use DCs with the respect to safety and neoantigen-specific T-cell induction ability.

According to recent reports revealing the importance of neoantigen-specific CD4^+^ T cell induction (42, 43), we have added long peptides that were expected to bind to HLA class II molecules to short peptides that bind to HLA class I molecules in recent 5 cases. However, the significance of the combined use of long peptides and the types of T cells induced by long peptide-pulsed DCs remain unclear. In the case of Rec #8, although induction of neoantigen-specific CD8^+^ T cells by the short peptide was insufficient, we observed the marked induction of CD4^+^ T cells by one long peptide that was possibly contributed to drastic tumor shrinkage in this patient. Our TCR repertoire analysis demonstrated that the population induced by the long peptide comprised CD4^+^ T cells, suggesting the importance of neoantigen-specific CD4^+^ T cells. While CD8^+^ T cells undoubtedly play a pivotal role in antitumor immunity, this result suggests that the induction of neoantigen-specific CD4^+^ T cells might be also important for vaccine therapy. Wolf et al. (44) recently reported that at least one CD4^+^ TCR-engineered T cell targeting one neoantigen is essential for maintenance of the efficacy of CD8^+^ TCR-engineered T cells together with antigen-presenting cells and cancer cells. Furthermore, Baleeiro et al. (42) reported the potential of HLA II neoantigen vaccines in pancreatic cancer based on the high expression of HLA-DR in pancreatic cancer cells. The antitumor effect of CD4^+^ T cells induced by long peptides binding to HLA class II is expected to have two different mechanisms (43, 45). One is the sustaining activation of CD8^+^ cytotoxic T lymphocytes (CTLs) via the CD40L-CD40 and CD27-CD70 pathways and the promotion of antibody production against B cells via DC activation, and the other is the direct cytotoxicity toward cancer cells. While an antigen-specific cytotoxicity can be expected in the case of high HLA class II expression on cancer cells, even if cancer cells do not express HLA class II, cytokine production such as IFNγor a nonspecific neoantigen-independent cytotoxicity may also be involved (46, 47). In the case of Rec #8, the absence of HLA-DR expression in cancer cells suggests an indirect mechanism of sustained activation of CD8^+^ CTLs or neoantigen-independent cytotoxic activity. Furthermore, in Rec#8, aggregations of B cells which are regarded to be tertiary lymphoid structures (TLSs), was observed within the tumor, suggesting that activation of neoantigen-specific helper T cells may have already occurred before the vaccinations. Further case numbers and studies are needed to assess the relationship between TLSs and induction of neoantigen-specific T cells by Neo-P DC vaccine. We recently reported that HLA class-I and -II hybrid neoantigen long peptide, namely, a Class-II neoantigen long peptide encapsulating a Class-I neoepitope (22). The hybrid neoantigen long peptides once incorporated into DCs, are processed intracellularly to present neoantigens to CD8^+^ T cells. In Case #8, the most strongly reacting long peptide (pep 6) encapsulated the class-I short neoepitope (pep 1), but the short peptide did not induce an immune response (**Figures 4C, T**; **Supplementary Tables 1**, **3**). Overall, these results suggest that the mechanism of the antitumor effect of long peptide-reactive CD4^+^ T cells is complex and requires further study.

The regulatory approval process for personalized vaccines is not so easy because peptides for each patient is personalized due to individual patient’s specific tumor mutations. Although our method is widely used for DC vaccines, this individualized approach requires meticulous validation and strict quality control of peptide production to ensure the safety, purity, and efficacy of the vaccines. However, recent advances in automation and bioprocessing technologies are being explored to overcome these challenges and streamline the production process. In addition, several clinical trials have confirmed the safety and immunogenicity of Neo-P DC vaccines (48, 49), supporting their potential for clinical application.

In this study, we used intranodal administration to elicit immunity, as we previously reported (20). While this method offers immunological advantages, it is also a practical challenge in clinical application, as it requires skilled personnel. To address these challenges, the involvement of experienced instructors and future studies on the administration route will be essential. By refining techniques and developing standardized protocols, Neo-P DC vaccine administration can be feasibly integrated into clinical practice.

The main limitation of this study is the small number of cases analyzed. An increase in the number of cases analyzed is needed in future research. Additionally, this retrospective analysis did not apply any specific patient selection criteria. However, since the number of patients is too small, we cannot exclude the possibility of unexpected selection bias. Hence, we need prospective studies to validate our findings. Nevertheless, in this preliminary retrospective study, the potential efficacy of the Neo-P DC vaccine against pancreatic cancer revealed in this study provides a strong rationale for progressing toward clinical trials to evaluate the clinical impact of the Neo-P DC vaccine in patients with pancreatic cancer. We are hopeful that this study will contribute to the development of new therapy that could represent a breakthrough in the currently challenging state of pancreatic cancer treatment.

## Data Availability

The data of TCR repertoire in the study are deposited in Sequence Read Archive (SRA), accession number PRJNA1244550. The human sequence data generated in this study are not publicly available due to patient privacy requirements but are available upon reasonable request from the corresponding author. Other data that support the findings of this study are available from the corresponding author upon reasonable request.
