# Supplementary material for: Neoantigen peptide-pulsed dendritic cell vaccine therapy after surgical treatment of pancreatic cancer: a retrospective study
Source: Front Immunol. 2025 Apr 3;16:1571182. doi: 10.3389/fimmu.2025.1571182 (PMC12004129; doi:10.3389/fimmu.2025.1571182)
Supplement: Supplementary file 1 [file DataSheet1.pdf]

**Supplementary table 1. The list of short neoantigen-peptides selected for MHC I in each recurrence cases**

Recurrence #1

| Peptide No. | gene | amino_acid | length | pos | peptide_mut | affinity_mut(nM) | peptide_wt | affinity_wt(nM) | HLA | tumor_exome(ref,var,freq) | normal_exome(ref,var,freq) |
|---|---|---|---|---|---|---|---|---|---|---|---|
| 1 | SLC35G6 | S271G | 10 | 5 | FTCVGYAVTK | 57 | FTCVSYAVTK | 45 | HLA-A11:01 | 145,19,0.12 | 133,2,0.01 |
| 2 | TARBP1 | I418F | 10 | 2 | FFIGPLMDAL | 77 | FIIGPLMDAL | 572 | HLA-C07:02 | 34,4,0.11 | 42,0,0.00 |
| 3 | TMEM41A | T112N | 10 | 8 | LLLCCVLNSV | 384 | LLLCCVLTSV | 296 | HLA-A02:07 | 53,6,0.102 | 146,0,0.000 |
| 4 | KCTD14 | T235M | 10 | 9 | KVFSKFYLMY | 29 | KVFSKFYLTY | 27 | HLA-A11:01 | 35,4,0.103 | 136,0,0.000 |
| 5 | SVEP1 | E2206A | 8 | 5 | VSCGAPPK | 33 | VSCGEPPK | 45 | HLA-A11:01 | 33,5,0.132 | 163,0,0.000 |
| 6 | RREB1 | M1370L | 8 | 5 | ATKLLDFK | 37 | ATKLMDFK | 34 | HLA-A11:01 | 108,12,0.100 | 248,0,0.000 |
| 7 | NUSAP1 | Q405K | 11 | 4 | KTYKKPHLQTK | 97 | KTYKQPHLQTK | 149 | HLA-A11:01 | 15,4,0.211 | 95,0,0.000 |
| 8 | MFAP3 | E201K | 10 | 7 | KLQKAFKIAK | 106 | KLQKAFEIAK | 93 | HLA-A11:01 | 53,6,0.102 | 104,0,0.000 |
| 9 | LRBA | I1375R | 8 | 8 | MVMACGGR | 144 | MVMACGGI | 14230 | HLA-A11:01 | 135,28,0.172 | 203,0,0.000 |
| 10 | VPS28 | V132M | 11 | 1 | MVSLFITVMDK | 157 | VVSLFITVMDK | 238 | HLA-A11:01 | 49,6,0.109 | 139,0,0.000 |
| 11 | KRAS | G12D | 9 | 5 | VVGADGVGK | 194 | VVGAGGVGK | 107 | HLA-A11:01 | 28,9,0.243 | 66,0,0.000 |
| 12 | DEF8 | R277H | 10 | 2 | YHCAECRAPI | 244 | YRCAECRAPI | 5252 | HLA-B38:02 | 28,4,0.125 | 173,0,0.000 |
| 13 | RIPK1 | E427K | 8 | 8 | FAQQRPYK | 282 | FAQQRPYE | 20549 | HLA-A11:01 | 34,4,0.105 | 135,0,0.000 |

Recurrence #2

| | gene | amino_acid | length | pos | peptide_mut | affinity_mut(nM) | peptide_wt | affinity_wt(nM) | HLA | tumor_exome(ref,var,freq) | normal_exome(ref,var,freq) |
|---|---|---|---|---|---|---|---|---|---|---|---|
| 1 | MXRA5 | T1961S | 8 | 5 | YQDVSVYL | 18 | YQDVTVYL | 16 | HLA-B39:01 | 247,54,0.18 | 266,0,0.00 |
| 2 | LRRC14 | C285S | 9 | 8 | AQMGRFTSL | 23 | AQMGRFTCL | 44 | HLA-B39:01 | 221,53,0.19 | 224,0,0.00 |
| 3 | MPPE1 | K276R | 8 | 2 | FRENYDVL | 29 | FKENYDVL | 128 | HLA-B39:01 | 240,63,0.21 | 237,0,0.00 |
| 4 | MEGF6 | R443Q | 11 | 10 | FQCSCEAGYQL | 32 | FQCSCEAGYRL | 55 | HLA-B39:01 | 97,48,0.33 | 88,0,0.00 |
| 5 | SC5D | I250F | 9 | 9 | QYFTLWDRF | 50 | QYFTLWDRI | 62 | HLA-A24:02 | 110,32,0.23 | 151,0,0.00 |
| 6 | TUBB4B | V258F | 10 | 2 | MFPFPRLHFF | 74 | MVPFPRLHFF | 699 | HLA-A24:02 | 330,63,0.16 | 350,0,0.00 |
| 7 | ZNF530 | R40C | 10 | 3 | LYCDVMLENF | 88 | LYRDVMLENF | 139 | HLA-A24:02 | 307,136,0.31 | 341,0,0.00 |
| 8 | IQCC | P364L | 11 | 8 | CYSKSGPLSSI | 117 | CYSKSGPPSSI | 130 | HLA-A24:02 | 183,36,0.16 | 211,0,0.00 |

Recurrence #3

| | gene | amino_acid | length | pos | peptide_mut | affinity_mut(nM) | peptide_wt | affinity_wt(nM) | HLA | tumor_exome(ref,var,freq) | normal_exome(ref,var,freq) |
|---|---|---|---|---|---|---|---|---|---|---|---|
| 1 | KRAS | G12V | 9 | 5 | VVGAVGVGK | 50 | VVGAGGVGK | 107 | HLA-A11:01 | 58,29,0.333 | 93,0,0.000 |
| 2 | ZNF354B | L509I | 8 | 3 | SSISNHQR | 72 | SSLSNHQR | 104 | HLA-A11:01 | 148,38,0.204 | 127,0,0.000 |
| 3 | ABCC11 | R69S | 9 | 3 | ALSTMIPFR | 76 | ALRTMIPFR | 1696 | HLA-A11:01 | 28,5,0.152 | 38,0,0.000 |
| 4 | TP53 | R248W | 10 | 9 | SSCMGGMNWR | 169 | SSCMGGMNRR | 204 | HLA-A11:01 | 65,53,0.449 | 232,0,0.000 |
| 5 | PGBD5 | V511A | 9 | 8 | AQFGERLAR | 223 | AQFGERLVR | 312 | HLA-A11:01 | 143,109,0.433 | 179,0,0.000 |
| 6 | STX1A | R246H | 10 | 6 | STRARHAGRK | 270 | STRARRAGRK | 261 | HLA-A11:01 | 67,57,0.460 | 210,0,0.000 |
| 7 | KIAA1407 | S794G | 8 | 5 | RSQEGLAR | 444 | RSQESLAR | 193 | HLA-A11:01 | 145,38,0.208 | 135,0,0.000 |
| 8 | IGF2BP1 | A40T | 10 | 9 | QFLVKSGYTF | 183 | QFLVKSGYAF | 635 | HLA-A24:02 | 170,110,0.393 | 278,0,0.000 |
| 9 | CNGA4 | R469H | 9 | 6 | MEEKGHEIL | 4 | MEEKGREIL | 11 | HLA-B40:01 | 223,155,0.410 | 321,0,0.000 |
| 10 | DNAH6 | T456M | 9 | 9 | IENTMHILM | 76 | IENTMHILT | 5495 | HLA-B40:01 | 154,41,0.210 | 154,0,0.000 |
| 11 | BEND5 | K392I | 10 | 2 | SIVNKYICEK | 26 | SKVNKYICEK | 8217 | HLA-A11:01 | 117,26,0.182 | 126,0,0.000 |
| 12 | ARSF | E252V | 9 | 2 | HVITEQPMK | 47 | HEITEQPMK | 15409 | HLA-A11:01 | 87,43,0.331 | 82,0,0.000 |

Recurrence #4

| No. | gene | amino_acid | length | pos | peptide_mut | affinity_mut(nM) | peptide_wt | affinity_wt(nM) | HLA | tumor_exome(ref,var,freq) | normal_exome(ref,var,freq) |
|---|---|---|---|---|---|---|---|---|---|---|---|
| 1 | UBE3B | H950Y | 9 | 2 | SYRVIIWLW | 21 | SHRVIIWLW | 1226 | HLA-A24:02 | 31,4,0.114 | 103,0,0.000 |
| 2 | DAGLB | G290W | 11 | 11 | HYMQFAAAAYW | 27 | HYMQFAAAAYG | 3849 | HLA-A24:02 | 30,4,0.118 | 58,0,0.000 |
| 3 | RPS3A | Q157P | 11 | 4 | SYAPHQQVRQI | 105 | SYAQHQQVRQI | 95 | HLA-A24:02 | 44,10,0.185 | 62,0,0.000 |
| 4 | TFEB | L186M | 9 | 1 | MSSSHLNVY | 117 | LSSSHLNVY | 684 | HLA-B15:18 | 10,4,0.286 | 38,0,0.000 |
| 5 | CYTH1 | D88Y | 11 | 2 | NYLLKNTCEDI | 167 | NDLLKNTCEDI | 30353 | HLA-A24:02 | 32,4,0.111 | 56,0,0.000 |

Recurrence #5

| No. | gene | amino_acid | length | pos | peptide_mut | affinity_mut(nM) | peptide_wt | affinity_wt(nM) | HLA | tumor_exome(ref,var,freq) | normal_exome(ref,var,freq) |
|---|---|---|---|---|---|---|---|---|---|---|---|
| 1 | ADAMTS5 | G675S | 11 | 6 | RAKGTSYYVVF | 30 | RAKGTGYYVVF | 36 | HLA-B15:01 | 55,16,0.225 | 160,0,0.000 |
| 2 | SOGA2 | T467M | 8 | 5 | RLERMVER | 32 | RLERTVER | 47 | HLA-A31:01 | 345,40,0.104 | 306,0,0.000 |
| 3 | ZMYM3 | P1338L | 11 | 2 | ILMDRSMLESM | 38 | IPMDRSMLESM | 10136 | HLA-B15:01 | 109,19,0.148 | 193,0,0.000 |
| 4 | LNX2 | A639S | 10 | 9 | KTIVLGTPSY | 42 | KTIVLGTPAY | 59 | HLA-B15:01 | 33,4,0.108 | 75,0,0.000 |
| 5 | BMP7 | A350T | 9 | 2 | ITPEGYAAY | 43 | IAPEGYAAY | 880 | HLA-A26:01 | 71,15,0.174 | 121,1,0.008 |

Recurrence #6

| | gene | amino_acid | length | pos | peptide_mut | affinity_mut(nM) | peptide_wt | affinity_wt(nM) | HLA | tumor_exome(ref,var,freq) | normal_exome(ref,var,freq) |
|---|---|---|---|---|---|---|---|---|---|---|---|
| 1 | EEA1 | R1038M | 10 | 10 | KQLQSDFYGM | 14 | KQLQSDFYGR | 752 | HLA-A02:06 | 24,4,0.143 | 66,0,0.000 |
| 2 | CCNYL1 | Q189K | 8 | 1 | KILKDITV | 14 | QILKDITV | 60 | HLA-A02:06 | 33,4,0.108 | 89,0,0.000 |
| 3 | BRWD3 | P1518Q | 8 | 2 | GQFSSSSF | 15 | GPFSSSSF | 3785 | HLA-B15:01 | 45,6,0.118 | 57,0,0.000 |
| 4 | OBSCN | K3875N | 8 | 3 | TVNALPAK | 23 | TVKALPAK | 66 | HLA-A11:01 | 42,6,0.125 | 48,0,0.000 |
| 5 | WDR33 | G141V | 9 | 9 | RLVTGASSV | 29 | RLVTGASSG | 7422 | HLA-A02:06 | 36,4,0.100 | 67,0,0.000 |
| 6 | ABCD4 | R136L | 10 | 7 | YTLNVLLDDI | 36 | YTLNVLRDDI | 292 | HLA-A02:06 | 47,6,0.113 | 63,0,0.000 |
| 7 | PDZD2 | G1222W | 8 | 1 | WQQPMTEL | 46 | GQQPMTEL | 206 | HLA-A02:06 | 55,8,0.127 | 75,0,0.000 |
| 8 | WAPAL | R731S | 8 | 7 | YILSRDSL | 47 | YILSRDRL | 232 | HLA-A02:06 | 36,4,0.100 | 76,0,0.000 |
| 9 | SYNE2 | L549F | 11 | 11 | FQKCGEIYKNF | 50 | FQKCGEIYKNL | 883 | HLA-B15:01 | 28,4,0.125 | 48,0,0.000 |
| 10 | CUTC | K254N | 10 | 4 | YSLNVTDVTK | 50 | YSLKVTDVTK | 88 | HLA-A11:01 | 21,4,0.160 | 61,0,0.000 |

Recurrence #7

| No. | gene | amino_acid | length | pos | peptide_mut | affinity_mut(nM) | peptide_wt | affinity_wt(nM) | HLA | tumor_exome(ref,var,freq) | normal_exome(ref,var,freq) |
|---|---|---|---|---|---|---|---|---|---|---|---|
| 1 | WHSC1L1 | C952Y | 11 | 11 | MPEGCWNCNDY | 6 | MPEGCWNCNDC | 2155 | HLA-B35:01 | 54,6,0.100 | 114,0,0.000 |
| 2 | EP300 | A467T | 9 | 8 | DPSSIERTY | 33 | DPSSIERAY | 12 | HLA-B35:01 | 42,5,0.106 | 66,0,0.000 |
| 3 | RP1 | S616L | 9 | 9 | ISADATHFL | 53 | ISADATHFS | 3764 | HLA-A02:06 | 73,15,0.170 | 123,0,0.000 |
| 4 | KRAS | Q61R | 8 | 8 | DILDTAGR | 91 | DILDTAGQ | 27682 | HLA-A33:03 | 72,8,0.100 | 99,0,0.000 |

Recurrence #8

| | gene | amino_acid | length | pos | peptide_mut | affinity_mut(nM) | peptide_wt | affinity_wt(nM) | HLA | tumor_exome(ref,var,freq) | normal_exome(ref,var,freq) |
|---|---|---|---|---|---|---|---|---|---|---|---|
| 1 | ARHGAP12 | G495V | 10 | 10 | WLSSWAVLQV | 17 | WLSSWAVLQG | 4838 | HLA-A02:01 | 120,8,0.062 | 142,0,0.000 |
| 2 | PRRC2C | S2260Y | 9 | 6 | KAWENYPNV | 23 | KAWENSPNV | 22 | HLA-A02:01 | 121,9,0.069 | 128,0,0.000 |
| 3 | ETV6 | V345I | 9 | 6 | LLWDYIYQL | 2 | LLWDYVYQL | 2 | HLA-A02:01 | 65,6,0.085 | 95,0,0.000 |
| 4 | HOXA5 | F5I | 8 | 1 | IVNSFCGR | 21 | FVNSFCGR | 15 | HLA-A33:03 | 113,12,0.096 | 131,0,0.000 |
| 5 | MUC16 | P14341L | 9 | 2 | QLGTTNYQR | 312 | QPGTTNYQR | 3507 | HLA-A33:03 | 73,4,0.052 | 148,0,0.000 |

Recurrence #9

| No. | gene | amino_acid | length | pos | peptide_mut | affinity_mut(nM) | peptide_wt | affinity_wt(nM) | HLA | tumor_exome(ref,var,freq) | normal_exome(ref,var,freq) |
|---|---|---|---|---|---|---|---|---|---|---|---|
| 1 | RBM47 | A495T | 11 | 10 | LQKNAAAAATM | 21 | LQKNAAAAAAM | 21 | HLA-B15:01 | 591,87,0.128 | 514,0,0.000 |
| 2 | KRAS | G12V | 9 | 5 | VVGAVGVGK | 50 | VVGAGGVGK | 107 | HLA-A11:01 | 38,17,0.309 | 175,0,0.000 |
| 3 | TMEM67 | A621T | 10 | 4 | ALKTLQFLHK | 74 | ALKALQFLHK | 64 | HLA-A11:01 | 165,37,0.183 | 565,0,0.000 |
| 4 | FLNA | R301W | 8 | 5 | MVKKWAEF | 85 | MVKKRAEF | 107 | HLA-B15:01 | 694,335,0.326 | 769,0,0.000 |
| 5 | EIF3H | D226G | 9 | 5 | SAVAGKHEL | 3 | SAVADKHEL | 4 | HLA-C03:03 | 112,34,0.233 | 290,0,0.000 |
