# Supplementary material for: Neoantigen peptide-pulsed dendritic cell vaccine therapy after surgical treatment of pancreatic cancer: a retrospective study
Source: Front Immunol. 2025 Apr 3;16:1571182. doi: 10.3389/fimmu.2025.1571182 (PMC12004129; doi:10.3389/fimmu.2025.1571182)
Supplement: Supplementary file 2 [file DataSheet2.pdf]

Supplementary table 2. The list of short neoantigen-peptides for MHC I selected in each adjuvant cases

Adjuvant #1

Peptide No.	gene	amino_acid	length	pos	peptide_mut	affinity_mut(nM)	peptide_wt	affinity_wt(nM)	HLA	tumor_exome(ref,var,freq)	normal_exome(ref,var,freq)
1	HCFC2	R107C	8	7	YELQASCW	20	YELQASRW	14	HLA-B44:03	30,4,0.12	44,0,0.00
2	HCFC2	R107C	11	5	ELQASCWLWKK	499	ELQASRWLWKK	288	HLA-A33:03	30,4,0.12	44,0,0.00
3	MUC6	V2046I	8	2	TIPPPTTL	193	TVPPPTTL	141	HLA-C14:03	47,9,0.16	40,0,0.00

Adjuvant #2

Peptide No.	gene	amino_acid	length	pos	peptide_mut	affinity_mut(nM)	peptide_wt	affinity_wt(nM)	HLA	tumor_exome(ref,var,freq)	normal_exome(ref,var,freq)
1	CNTN1	V168L	10	9	RWLLNEFPLF	14	RWLLNEFPVF	20	HLA-A24:02	112,64,0.36	214,0,0.00
2	ARPC1A	G25W	9	6	IYKKNWSQW	18	IYKKNGSQW	178	HLA-A24:02	51,6,0.11	86,0,0.00
3	SLC25A38	G106C	9	4	IYFCTLYSL	42	IYFGTLYSL	51	HLA-A24:02	33,4,0.11	84,0,0.00
4	LILRB3	G565V	10	10	MASPPSSLSV	16	MASPPSSLSG	3098	HLA-C12:02	35,4,0.10	65,0,0.00
5	SLC25A3	L179M	10	7	FFADIAMAPM	17	FFADIALAPM	23	HLA-C12:02	28,4,0.13	45,0,0.00
6	F5	R538M	11	1	MAADIEQQAVF	45	RAADIEQQAVF	174	HLA-C12:02	21,4,0.16	73,0,0.00
7	ABCF2	G409V	9	5	YTKDVPCIY	71	YTKDGPCIY	94	HLA-C12:02	26,4,0.13	33,0,0.00

Adjuvant #3

Peptide No.	gene	amino_acid	length	pos	peptide_mut	affinity_mut(nM)	peptide_wt	affinity_wt(nM)	HLA	tumor_exome(ref,var,freq)	normal_exome(ref,var,freq)
1	OR4K15	L64F	10	7	LYLAILFGNF	34	LYLAILLGNF	65	HLA-A24:02	40,5,0.111	87,0,0.000
2	BSX	P72H	11	1	HLHKGDHHHPY	32	PLHKGDHHHPY	958	HLA-B35:01	48,6,0.111	121,0,0.000
3	TRIP12	P1240H	9	9	FPVKVHDFH	36	FPVKVHDFP	1460	HLA-B35:01	54,6,0.100	78,0,0.000
4	SLC13A5	G428W	9	4	EASWLSVWM	44	EASGLSVWM	96	HLA-B35:01	32,4,0.111	66,0,0.000
5	NOTCH4	S1105Y	11	11	HCHHGGLCLPY	52	HCHHGGLCLPS	12661	HLA-B35:01	51,6,0.105	58,0,0.000
6	HLA-DRB	S24F	9	5	MVLSFPLAL	82	MVLSSPLAL	73	HLA-B35:01	35,6,0.146	99,0,0.000
7	HS6ST1	R249S	9	5	LANNSQVRM	90	LANNRQVRM	745	HLA-B35:01	64,29,0.312	51,0,0.000
8	CCDC17	G395W	11	2	PWAGLVIFYDF	101	PGAGLVIFYDF	8834	HLA-A24:02	36,4,0.100	103,0,0.000
9	TUT1	G462V	11	3	LSVSGPLLSNY	321	LSGSGPLLSNY	2038	HLA-B35:01	43,5,0.104	73,0,0.000
10	LEPREL1	D491Y	11	11	SVASGIMLVGY	329	SVASGIMLVGD	30471	HLA-B35:01	32,4,0.111	81,0,0.000

Adjuvant #4

Peptide No.	gene	amino_acid	length	pos	peptide_mut	affinity_mut(nM)	peptide_wt	affinity_wt(nM)	HLA	tumor_exome(ref,var,freq)	normal_exome(ref,var,freq)
1	ENTPD5	H23N	9	3	VSNRNQQTW	6	VSHRNQQTW	9	HLA-B58:01	23,4,0.148	54,0,0.000
2	STRIP1	P423Q	9	8	LTCPKGLQW	12	LTCPKGLPW	19	HLA-B58:01	25,4,0.138	59,0,0.000
3	FCAR	L115M	9	5	YSDTMELVV	15	YSDTLELVV	19	HLA-A01:01	20,4,0.167	90,0,0.000
4	LMO7	R1051W	9	5	AEIEWETSV	107	AEIERETSV	448	HLA-B40:06	22,4,0.154	44,0,0.000
5	HMGXB3	L613F	9	9	YSCTVTLDF	83	YSCTVTLDL	1458	HLA-B58:01	36,4,0.100	54,0,0.000
6	CBLN4	G152W	9	9	KPVISAFAW	158	KPVISAFAG	21263	HLA-B58:01	39,5,0.114	79,0,0.000
7	ENTPD5	H23N	10	4	AVSNRNQQTW	203	AVSHRNQQTW	94	HLA-B58:01	23,4,0.148	54,0,0.000
8	COL18A1	G1361V	8	5	RGIRVADF	304	RGIRGADF	1592	HLA-B58:01	28,4,0.125	67,0,0.000

Adjuvant #5

Peptide No.	gene	amino_acid	length	pos	peptide_mut	affinity_mut(nM)	peptide_wt	affinity_wt(nM)	HLA	tumor_exome(ref,var,freq)	normal_exome(ref,var,freq)
1	CCNB2	V191I	11	5	LYMCIGIMDRF	30	LYMCVGIMDRF	34	HLA-A24:02	33,4,0.108	66,0,0.000
2	LSMEM2	E15K	11	1	KETQEDSVAPM	150	EETQEDSVAPM	993	HLA-B40:02	101,12,0.106	203,0,0.000
3	GRID2IP	R578Q	11	1	QASPPGPSPAV	56	RASPPGPSPAV	42	HLA-C03:03	76,22,0.224	147,0,0.000
4	STOX1	K451N	11	7	GSIRLENHPKL	385	GSIRLEKHPKL	419	HLA-C03:03	85,10,0.105	123,0,0.000

Adjuvant #6

Peptide No.	gene	amino_acid	length	pos	peptide_mut	affinity_mut(nM)	peptide_wt	affinity_wt(nM)	HLA	tumor_exome(ref,var,freq)	normal_exome(ref,var,freq)
1	ATP11B	V347F	9	1	FLYNFIIPI	2	VLYNFIIPI	5	HLA-A02:01	35,4,0.103	97,0,0.000
2	TADA2A	D58N	10	7	YKKHQSNHTY	134	YKKHQSDHTY	158	HLA-B15:01	23,4,0.148	89,0,0.000
3	ACACB	E1884K	10	3	HIKEGGESRY	151	HIEEGGESRY	1722	HLA-B15:01	23,4,0.148	57,0,0.000
4	HERPUD1	P313L	10	4	RPRLVQNFPN	153	RPRPVQNFPN	113	HLA-B07:02	35,4,0.103	67,0,0.000
5	SCGB1D2	R74Q	11	5	SLQKQSLIAEV	120	SLQKRSLIAEV	142	HLA-A02:01	33,4,0.108	82,0,0.000
6	DSC3	L396F	9	9	NWRVNFTIF	303	NWRVNFTIL	3334	HLA-A24:02	28,4,0.125	107,0,0.000

Adjuvant #7

Peptide No.	gene	amino_acid	length	pos	peptide_mut	affinity_mut(nM)	peptide_wt	affinity_wt(nM)	HLA	tumor_exome(ref,var,freq)	normal_exome(ref,var,freq)
1	MBOAT1	T353I	8	3	IQIATWLK	28	IQTATWLK	74	HLA-A11:01	91,6,0.062	94,0,0.000
2	CACNA1H	F1444I	9	4	FIIIGILGV	6	FIIFGILGV	5	HLA-A02:06	456,32,0.066	343,0,0.000
3	TACR3	I105L	8	7	AVLGNLLV	11	AVLGNLIV	29	HLA-A02:06	144,8,0.053	157,0,0.000
4	TGFBRAP	I529L	9	1	LVDFLTYCL	230	IVDFLTYCL	255	HLA-A02:06	63,5,0.074	103,0,0.000
