# Supplementary material for: Neoantigen peptide-pulsed dendritic cell vaccine therapy after surgical treatment of pancreatic cancer: a retrospective study
Source: Front Immunol. 2025 Apr 3;16:1571182. doi: 10.3389/fimmu.2025.1571182 (PMC12004129; doi:10.3389/fimmu.2025.1571182)
Supplement: Supplementary file 3 [file DataSheet3.pdf]

Supplementary table 3. The list of long neoantigen-peptides for MHC II selected in combined cases

Recurrence #7

Peptide No.	gene	amino_acid	length	pos	peptide_mut	affinity_mut(nM	peptide_wt	affinity_wt(nM)	HLA	tumor_var(rna)	tumor_exome(ref,var,freq)	normal_exome(ref,var,freq)
5	WHSC1L1	C952Y	17	1	YKAGKKLHYKQIVWVKL	11	CKAGKKLHYKQIVWVKL	11	DRB1:1201	38	54,6,0.100	114,0,0.000
6	EP300	A467T	16	6	SSIERTYAALGLPYQV	57	SSIERAYAALGLPYQV	61	DRB1:1201	97	42,5,0.106	66,0,0.000
7	KRAS	Q61R	15	1	REEYSAMRDQYMRTG	322	QEEYSAMRDQYMRTG	358	DRB1:1201	31	72,8,0.100	99,0,0.000

Recurrence #8

Peptide No.	gene	amino_acid	length	pos	peptide_mut	affinity_mut(nM	peptide_wt	affinity_wt(nM)	HLA	tumor_var(rna)	tumor_exome(ref,var,freq)	normal_exome(ref,var,freq)
6	ARHGAP12	G495V	18	18	NGKKVRKNWLSSWAVLQV	44	NGKKVRKNWLSSWAVLQG	56	DRB1:1501	105	120,8,0.062	142,0,0.000
7	PRRC2C	S2260Y	18	1	YPNVREKGSPVTSTAPPI	72	SPNVREKGSPVTSTAPPI	72	DRB1:1302	63	121,9,0.069	128,0,0.000
8	ETV6	V345I	18	14	IGRIADCRLLWDYIYQLL	34	IGRIADCRLLWDYVYQLL	35	DRB1:1501	0	65,6,0.085	95,0,0.000
9	HOXA5	F5I	17	5	MSSYIVNSFCGRYPNGP	210	MSSYFVNSFCGRYPNGP	298	DRB1:1501	0	113,12,0.096	131,0,0.000

Recurrence #9

Peptide No.	gene	amino_acid	length	pos	peptide_mut	affinity_mut(nM	peptide_wt	affinity_wt(nM)	HLA	tumor_var(rna)	tumor_exome(ref,var,freq)	normal_exome(ref,var,freq)
6	TMEM67	A621T	18	4	ALKTLQFLHKLISQITID	27	ALKALQFLHKLISQITID	27	DRB1:0405	71	165,37,0.183	565,0,0.000
7	EIF3H	D226G	18	2	AGKHELLSLASSNHLGKN	30	ADKHELLSLASSNHLGKN	29	DRB1:1501	299	112,34,0.233	290,0,0.000
8	KRAS	G12V	18	1	VGVGKSALTIQLIQNHFV	39	GGVGKSALTIQLIQNHFV	39	DRB1:0405	16	38,17,0.309	175,0,0.000
9	FLNA	R301W	18	11	IEPTGNMVKKWAEFTVET	99	IEPTGNMVKKRAEFTVET	352	DRB1:1501	591	694,335,0.326	769,0,0.000

Adjuvant #6

Peptide No.	gene	amino_acid	length	pos	peptide_mut	affinity_mut(nM	peptide_wt	affinity_wt(nM)	HLA	tumor_var(rna)	tumor_exome(ref,var,freq)	normal_exome(ref,var,freq)
7	TADA2A	D58N	18	15	QCFTRGFEYKKHQSNHTY	375	QCFTRGFEYKKHQSDHTY	862	DRB1:1501	-	23,4,0.148	89,0,0.000
8	ATP11B	V347F	17	1	FLYNFIIPISLYVTVEM	35	VLYNFIIPISLYVTVEM	32	DRB1:1501	-	35,4,0.103	97,0,0.000
9	DSC3	L396F	18	17	DKDLINTANWRVNFTIFK	80	DKDLINTANWRVNFTILK	95	DRB1:1501	-	28,4,0.125	107,0,0.000

Adjuvant #7

Peptide No.	gene	amino_acid	length	pos	peptide_mut	affinity_mut(nM	peptide_wt	affinity_wt(nM)	HLA	tumor_var(rna)	tumor_exome(ref,var,freq)	normal_exome(ref,var,freq)
5	MBOAT1	T353I	18	11	KMYLENWNIQIATWLKCV	24	KMYLENWNIQTATWLKCV	34	DRB1:0901	19	91,6,0.062	94,0,0.000
6	TGFBRAP1	I529L	18	4	YEYLVDFLTYCLDEELVW	39	YEYIVDFLTYCLDEELVW	41	DRB1:0405	0	63,5,0.074	103,0,0.000
