# Supplementary material for: Neoantigen peptide-pulsed dendritic cell vaccine therapy after surgical treatment of pancreatic cancer: a retrospective study
Source: Front Immunol. 2025 Apr 3;16:1571182. doi: 10.3389/fimmu.2025.1571182 (PMC12004129; doi:10.3389/fimmu.2025.1571182)
Supplement: Supplementary file 4 [file DataSheet4.pdf]

Supplementary figure S1

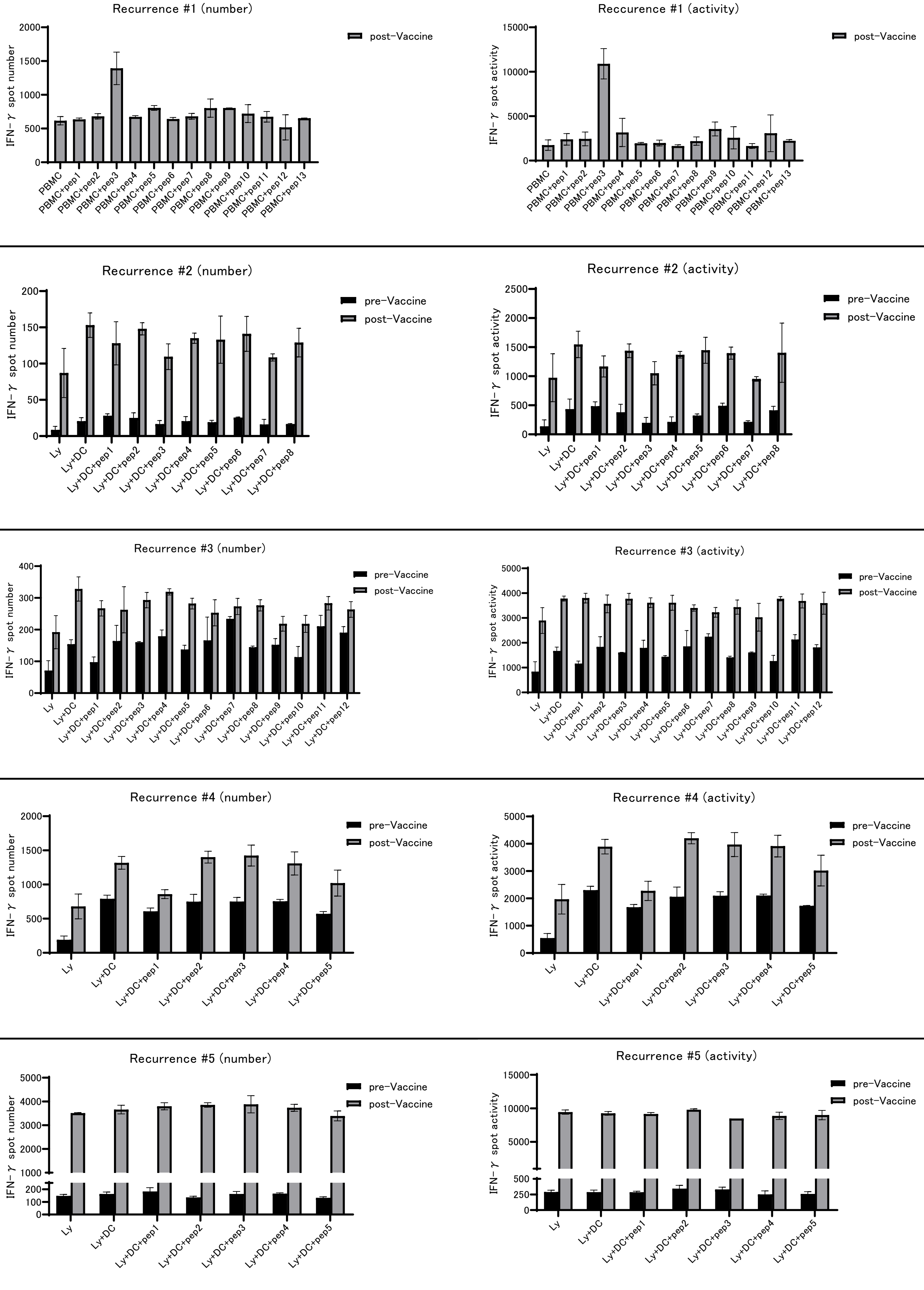

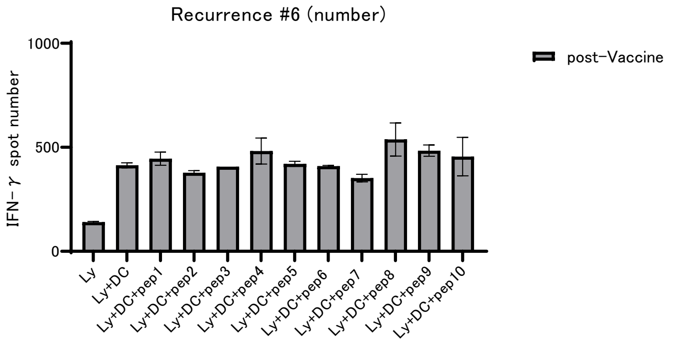
Recurrence #6 (number)
post-Vaccine
IFN-γ spot number
0
500
1000
Ly
Ly+DC
Ly+DC+pep1
Ly+DC+pep2
Ly+DC+pep3
Ly+DC+pep4
Ly+DC+pep5
Ly+DC+pep6
Ly+DC+pep7
Ly+DC+pep8
Ly+DC+pep9
Ly+DC+pep10

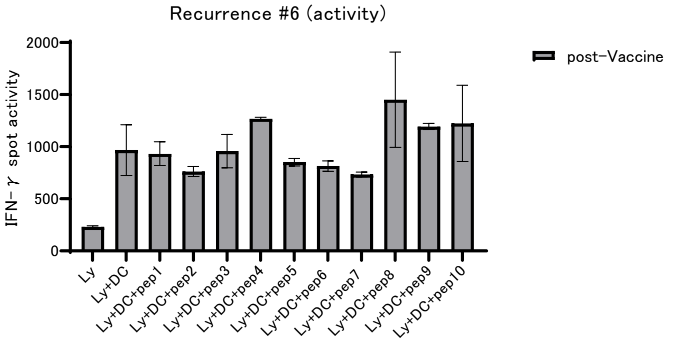
Recurrence #6 (activity)
post-Vaccine
IFN-γ spot activity
0
500
1000
1500
2000
Ly
Ly+DC
Ly+DC+pep1
Ly+DC+pep2
Ly+DC+pep3
Ly+DC+pep4
Ly+DC+pep5
Ly+DC+pep6
Ly+DC+pep7
Ly+DC+pep8
Ly+DC+pep9
Ly+DC+pep10

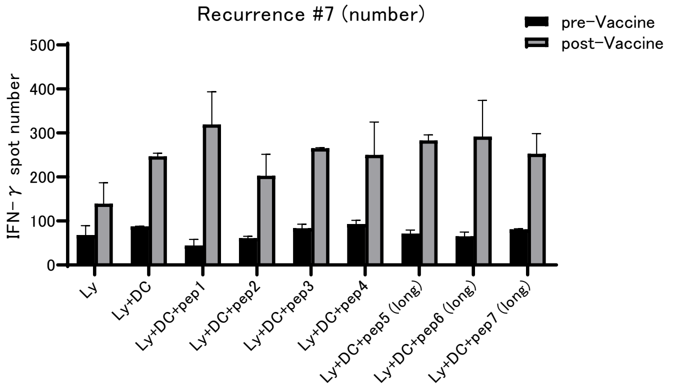
Recurrence #7 (number)
pre-Vaccine
post-Vaccine
IFN-γ spot number
0
100
200
300
400
500
Ly
Ly+DC
Ly+DC+pep1
Ly+DC+pep2
Ly+DC+pep3
Ly+DC+pep4
Ly+DC+pep5 (long)
Ly+DC+pep6 (long)
Ly+DC+pep7 (long)

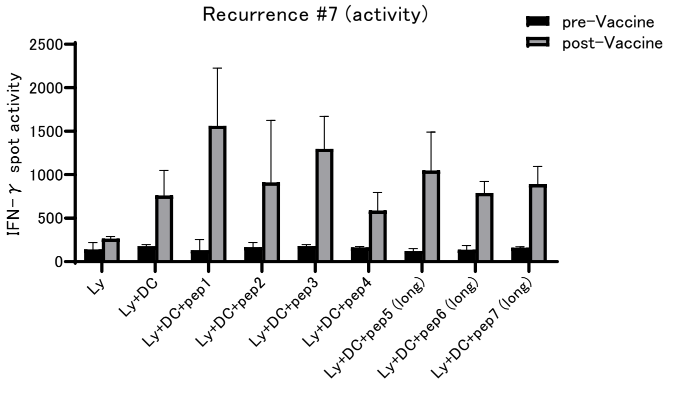
Recurrence #7 (activity)
pre-Vaccine
post-Vaccine
IFN-γ spot activity
0
500
1000
1500
2000
2500
Ly
Ly+DC
Ly+DC+pep1
Ly+DC+pep2
Ly+DC+pep3
Ly+DC+pep4
Ly+DC+pep5 (long)
Ly+DC+pep6 (long)
Ly+DC+pep7 (long)

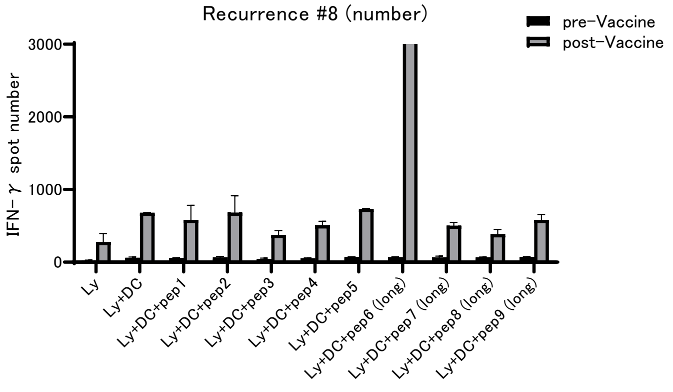
Recurrence #8 (number)
pre-Vaccine
post-Vaccine
IFN-γ spot number
0
1000
2000
3000
Ly
Ly+DC
Ly+DC+pep1
Ly+DC+pep2
Ly+DC+pep3
Ly+DC+pep4
Ly+DC+pep5
Ly+DC+pep6 (long)
Ly+DC+pep7 (long)
Ly+DC+pep8 (long)
Ly+DC+pep9 (long)

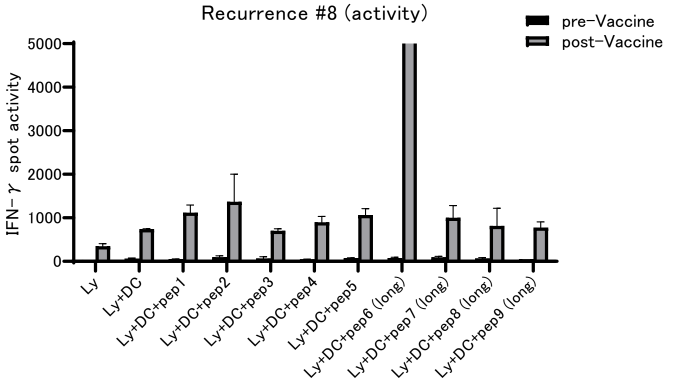
Recurrence #8 (activity)
pre-Vaccine
post-Vaccine
IFN-γ spot activity
0
1000
2000
3000
4000
5000
Ly
Ly+DC
Ly+DC+pep1
Ly+DC+pep2
Ly+DC+pep3
Ly+DC+pep4
Ly+DC+pep5
Ly+DC+pep6 (long)
Ly+DC+pep7 (long)
Ly+DC+pep8 (long)
Ly+DC+pep9 (long)

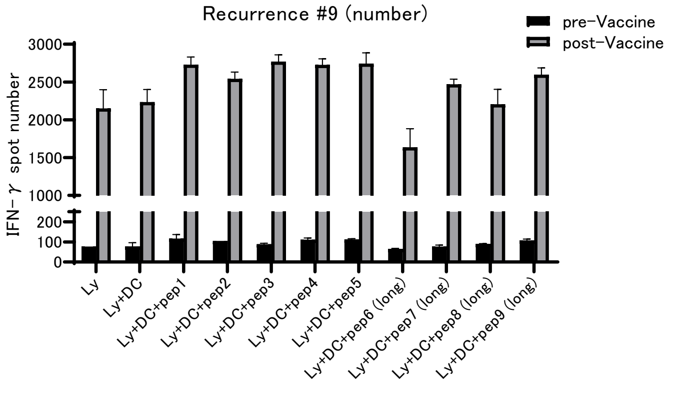
Recurrence #9 (number)
pre-Vaccine
post-Vaccine
IFN-γ spot number
0
100
200
1000
1500
2000
2500
3000
Ly
Ly+DC
Ly+DC+pep1
Ly+DC+pep2
Ly+DC+pep3
Ly+DC+pep4
Ly+DC+pep5
Ly+DC+pep6 (long)
Ly+DC+pep7 (long)
Ly+DC+pep8 (long)
Ly+DC+pep9 (long)

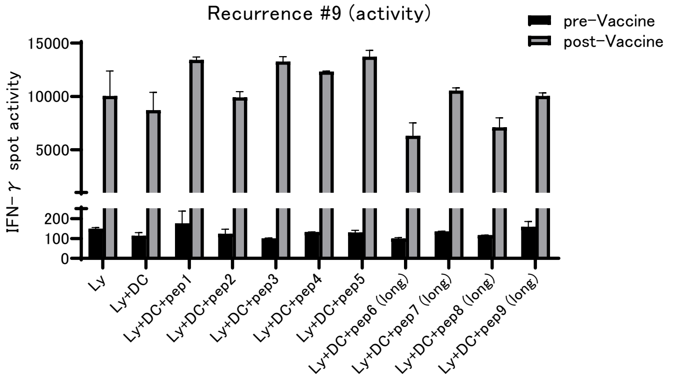
Recurrence #9 (activity)
pre-Vaccine
post-Vaccine
IFN-γ spot activity
0
100
200
5000
10000
15000
Ly
Ly+DC
Ly+DC+pep1
Ly+DC+pep2
Ly+DC+pep3
Ly+DC+pep4
Ly+DC+pep5
Ly+DC+pep6 (long)
Ly+DC+pep7 (long)
Ly+DC+pep8 (long)
Ly+DC+pep9 (long)

Supplementary Figure S1. The evaluation of neoantigen-specific T cells by IFN-γ ELISpot assay (recurrence cases)

Immune responses of peripheral blood lymphocytes to neoantigen peptides before and after Neo-P DC vaccine therapy in all recurrence cases. Each panel shows the IFN-γ ELISpot response to neoantigen peptide measured in peripheral blood lymphocytes from each patient [Ly, lymphocytes alone; Ly + DC, lymphocytes + dendritic cells; Ly + DC + pep (I), lymphocytes + dendritic cells + neoantigen peptide I (class I peptide); Ly + DC + pep (II), lymphocytes + dendritic cells + neoantigen peptide II (class II peptide).
