# Supplementary material for: Neoantigen peptide-pulsed dendritic cell vaccine therapy after surgical treatment of pancreatic cancer: a retrospective study
Source: Front Immunol. 2025 Apr 3;16:1571182. doi: 10.3389/fimmu.2025.1571182 (PMC12004129; doi:10.3389/fimmu.2025.1571182)
Supplement: Supplementary file 5 [file DataSheet5.pdf]

Supplementary figure S2

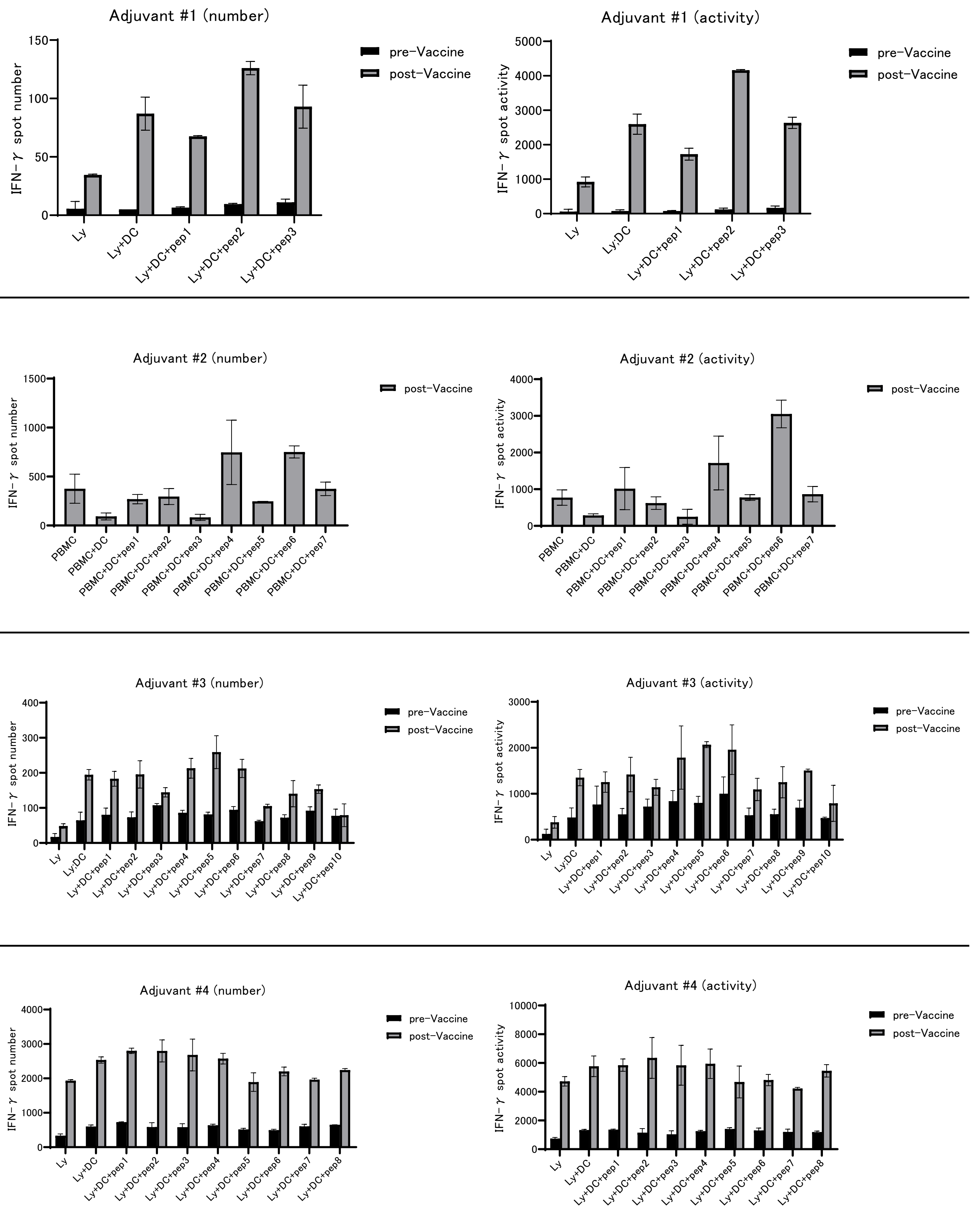

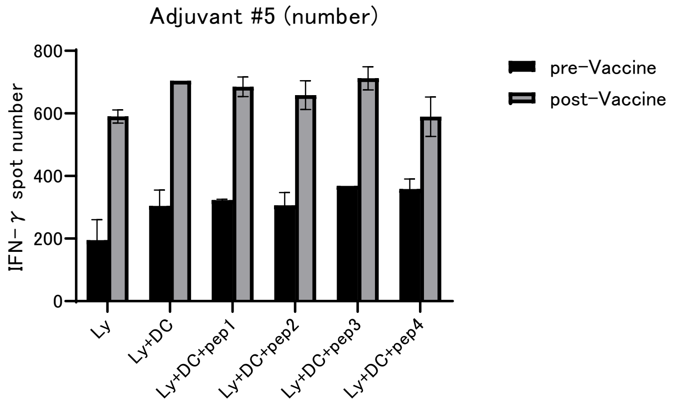
Adjuvant #5 (number)
IFN-γ spot number
800
600
400
200
0
Ly
Ly+DC
Ly+DC+pep1
Ly+DC+pep2
Ly+DC+pep3
Ly+DC+pep4
pre-Vaccine
post-Vaccine

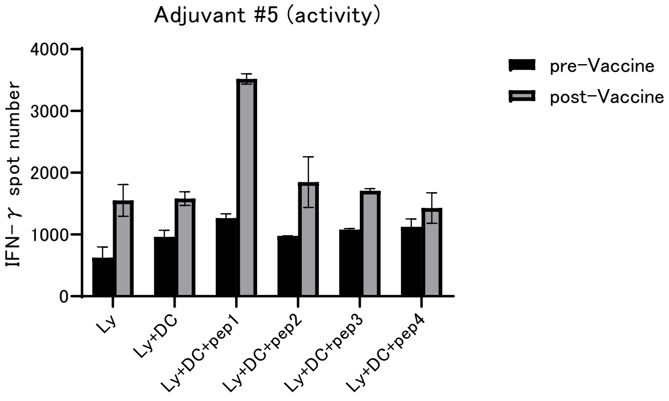
Adjuvant #5 (activity)
IFN-γ spot number
4000
3000
2000
1000
0
Ly
Ly+DC
Ly+DC+pep1
Ly+DC+pep2
Ly+DC+pep3
Ly+DC+pep4
pre-Vaccine
post-Vaccine

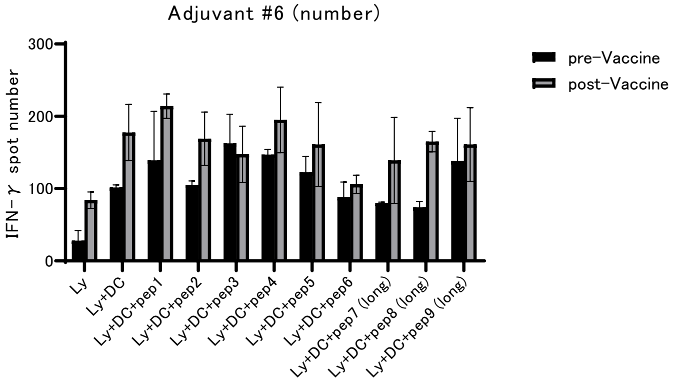
Adjuvant #6 (number)
IFN-γ spot number
300
200
100
0
Ly
Ly+DC
Ly+DC+pep1
Ly+DC+pep2
Ly+DC+pep3
Ly+DC+pep4
Ly+DC+pep5
Ly+DC+pep6
Ly+DC+pep7 (long)
Ly+DC+pep8 (long)
Ly+DC+pep9 (long)
pre-Vaccine
post-Vaccine

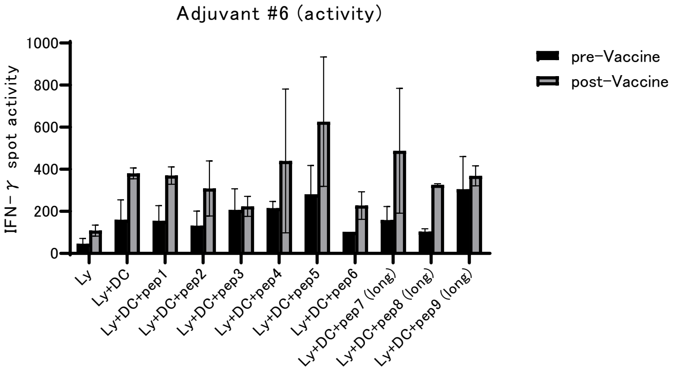
Adjuvant #6 (activity)
IFN-γ spot activity
1000
800
600
400
200
0
Ly
Ly+DC
Ly+DC+pep1
Ly+DC+pep2
Ly+DC+pep3
Ly+DC+pep4
Ly+DC+pep5
Ly+DC+pep6
Ly+DC+pep7 (long)
Ly+DC+pep8 (long)
Ly+DC+pep9 (long)
pre-Vaccine
post-Vaccine

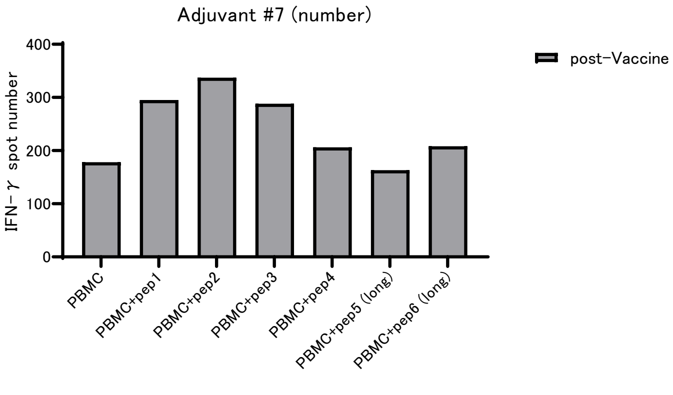
Adjuvant #7 (number)
IFN-γ spot number
400
300
200
100
0
PBMC
PBMC+pep1
PBMC+pep2
PBMC+pep3
PBMC+pep4
PBMC+pep5 (long)
PBMC+pep6 (long)
post-Vaccine

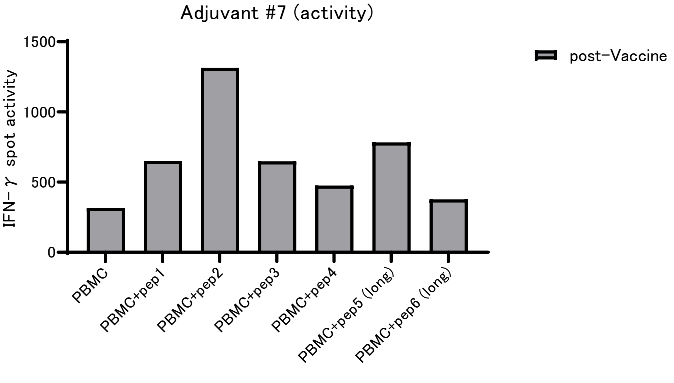
Adjuvant #7 (activity)
IFN-γ spot activity
1500
1000
500
0
PBMC
PBMC+pep1
PBMC+pep2
PBMC+pep3
PBMC+pep4
PBMC+pep5 (long)
PBMC+pep6 (long)
post-Vaccine

Supplementary Figure S2. The evaluation of neoantigen-specific T cells by IFN-γ ELISpot assay (adjuvant cases)

Immune responses of peripheral blood lymphocytes to neoantigen peptides before and after Neo-P DC vaccine therapy in all adjuvant cases. Each panel shows the IFN-γ ELISpot response to neoantigen peptide measured in peripheral blood lymphocytes from each patient [Ly, lymphocytes alone; Ly + DC, lymphocytes + dendritic cells; Ly + DC + pep (I), lymphocytes + dendritic cells + neoantigen peptide I (class I peptide); Ly + DC + pep (II), lymphocytes + dendritic cells + neoantigen peptide II (class II peptide).
